# 4-[(3-Benzamido­methyl-6-phenyl-6,7-dihydro-5*H*-1,2,4-triazolo[3,4-*b*][1,3,4]thia­diazin-7-yl)carbon­yl]-3-phenyl-1,2,3-oxadiazol-3-ium-5-olate 0.06-hydrate

**DOI:** 10.1107/S1600536810049998

**Published:** 2010-12-15

**Authors:** Hoong-Kun Fun, Madhukar Hemamalini, Nithinchandra, Balakrishna Kalluraya

**Affiliations:** a X-ray Crystallography Unit, School of Physics, Universiti Sains Malaysia, 11800 USM, Penang, Malaysia; b Department of Studies in Chemistry, Mangalore University, Mangalagangotri, Mangalore 574 199, India

## Abstract

The asymmetric unit of the title compound, C_27_H_21_N_7_O_4_S·0.06H_2_O, contains four syndone mol­ecules and a water mol­ecule with a site occupancy of 0.25. In two of the syndone mol­ecules, three atoms in a terminal phenyl ring are disordered over two sets of sites, with occupancy ratios of 0.500 (18):0.500 (18) and 0.512 (17):0.488 (17). The dihedral angles between terminal phenyl rings for the syndone mol­ecules are 23.3 (4), 45.57 (16), 68.46 (16) and 56.5 (3)°. In the crystal, mol­ecules are connected *via* N—H⋯N, N—H⋯O, O—H⋯O, O—H⋯N and C—H⋯O hydrogen bonds, forming a three-dimensional network.

## Related literature

For biological applications of sydnones, see: Newton & Ramsden (1982 ▶); Wagner & Hill (1974 ▶); Kalluraya & Rahiman (1997 ▶); Kalluraya *et al.* (2002 ▶). For the stability of the temperature controller used in the data collection, see: Cosier & Glazer (1986 ▶).
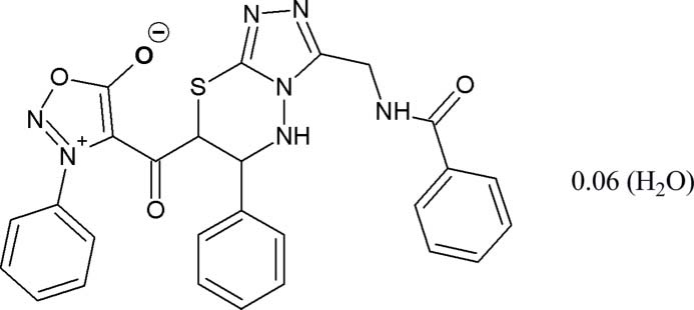


## Experimental

### 

#### Crystal data

C_27_H_21_N_7_O_4_S·0.06H_2_O
*M* *_r_* = 540.69
Triclinic, 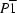

*a* = 15.6242 (19) Å
*b* = 18.7430 (19) Å
*c* = 18.9689 (19) Å
α = 111.235 (2)°
β = 93.970 (2)°
γ = 101.636 (2)°
*V* = 5009.4 (9) Å^3^
*Z* = 8
Mo *K*α radiation
μ = 0.18 mm^−1^
*T* = 100 K
0.64 × 0.08 × 0.07 mm

#### Data collection

Bruker APEXII DUO CCD area-detector diffractometer
Absorption correction: multi-scan (*SADABS*; Bruker, 2009) ▶ *T*_min_ = 0.893, *T*_max_ = 0.988
54193 measured reflections
17604 independent reflections
12624 reflections with *I* > 2σ(*I*)
*R*_int_ = 0.057

#### Refinement

*R*[*F* ^2^ > 2σ(*F* ^2^)] = 0.046
*wR*(*F* ^2^) = 0.141
*S* = 1.04
17604 reflections
1498 parameters
54 restraints
H atoms treated by a mixture of independent and constrained refinement
Δρ_max_ = 1.41 e Å^−3^
Δρ_min_ = −0.30 e Å^−3^

### 

Data collection: *APEX2* (Bruker, 2009 ▶); cell refinement: *SAINT* (Bruker, 2009 ▶); data reduction: *SAINT*; program(s) used to solve structure: *SHELXTL* (Sheldrick, 2008 ▶); program(s) used to refine structure: *SHELXTL*; molecular graphics: *SHELXTL*; software used to prepare material for publication: *SHELXTL* and *PLATON* (Spek, 2009 ▶).

## Supplementary Material

Crystal structure: contains datablocks global, I. DOI: 10.1107/S1600536810049998/hb5742sup1.cif

Structure factors: contains datablocks I. DOI: 10.1107/S1600536810049998/hb5742Isup2.hkl

Additional supplementary materials:  crystallographic information; 3D view; checkCIF report

## Figures and Tables

**Table 1:** Hydrogen-bond geometry (Å, °)

*D*—H⋯*A*	*D*—H	H⋯*A*	*D*⋯*A*	*D*—H⋯*A*
O1*W*—H2*W*1⋯O2*C*	0.85	1.92	2.772 (4)	175
O1*W*—H1*W*1⋯N6*B*	0.85	2.10	2.944 (4)	174
N7*A*—H1*NA*⋯N5*B*^i^	0.94 (5)	2.15 (5)	3.017 (4)	153 (4)
N7*A*—H1*NA*⋯N6*B*^i^	0.94 (5)	2.19 (6)	3.016 (4)	146 (3)
N2*A*—H2*NA*⋯O3*A*	0.78 (3)	2.35 (3)	2.912 (3)	130 (3)
N7*B*—H1*NB*⋯N6*A*^ii^	0.86 (4)	2.16 (4)	2.964 (4)	155 (3)
N3*B*—H2*NB*⋯O3*B*	0.88 (3)	2.25 (3)	2.865 (3)	127 (3)
N7*C*—H1*NC*⋯N6*D*^iii^	0.80 (3)	2.21 (3)	2.990 (4)	165 (3)
N3*C*—H2*NC*⋯O3*C*	0.91 (3)	2.21 (3)	2.895 (3)	132 (3)
N7*D*—H1*ND*⋯N6*C*^iii^	0.89 (3)	2.25 (3)	3.082 (4)	157 (3)
N3*D*—H2*ND*⋯O3*D*	0.94 (3)	2.07 (3)	2.841 (3)	138 (3)
C1*A*—H1*XA*⋯O2*B*^i^	1.16	2.43	3.471 (10)	148
C5*A*—H5*AA*⋯O4*A*^iv^	0.93	2.45	3.294 (4)	151
C1*B*—H1*BA*⋯O4*B*^v^	0.93	2.40	3.278 (4)	158
C11*A*—H11*A*⋯O4*A*^iv^	0.98	2.60	3.291 (4)	128
C5*B*—H5*BA*⋯O2*A*^ii^	0.93	2.51	3.405 (4)	162
C19*D*—H19*D*⋯O2*B*^vi^	0.93	2.41	3.289 (11)	157
C5*D*—H5*DA*⋯O4*D*^iii^	0.93	2.47	3.328 (4)	154
C23*A*—H23*A*⋯O3*A*^iv^	0.93	2.59	3.318 (4)	136
C23*B*—H23*B*⋯O3*B*^v^	0.93	2.41	3.240 (4)	149
C27*C*—H27*C*⋯O3*C*^vii^	0.93	2.52	3.359 (4)	150
C27*D*—H27*D*⋯O3*D*^iii^	0.93	2.42	3.258 (4)	149

Symmetry codes: (i) 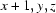; (ii) 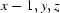; (iii) 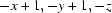; (iv) 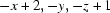; (v) 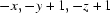; (vi) 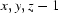; (vii) 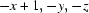.

**Fig. 1.**
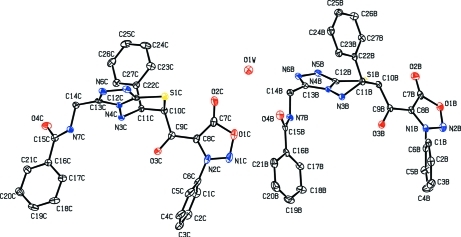
a. The undisordered syndone molecules in the title compound, showing 30% probability displacement ellipsoids [H atoms are omitted for clarity].

**Fig. 2.**
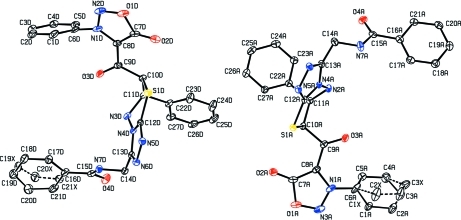
b. The disordered syndone molecules in the title compound, showing 30% probability displacement ellipsoids [H atoms are omitted for clarity]. Dotted lines represents disordered components.

**Fig. 3.**
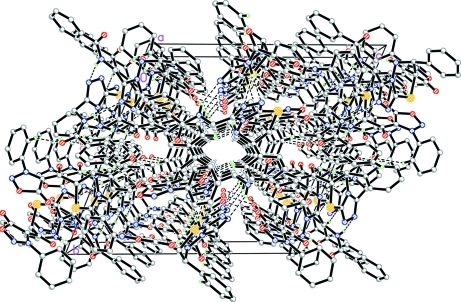
The crystal packing of the title compound (I).

## supplementary crystallographic information

### Comment

Sydnones are class of mesoionic compounds containing a 1,2,3-oxadiazole ring system. A number of sydnone derivatives have shown diverse biological activities such as anti-inflammatory, analgesic and anti-arthritic (Newton & Ramsden, 1982; Wagner & Hill, 1974) properties. Sydnones possessing heterocyclic moieties at the 4-position are also known for a wide range of biological properties (Kalluraya & Rahiman, 1997). Encouraged by these reports and in continuation of our research for biologically active nitrogen containing heterocycles, a triazolothiadiazole moiety at the 4-position of the phenylsydnone was introduced. A series of triazolothiadiazoles were synthesized by the reaction of 2,3-dibromo-1-(3'-arylsydnon-4'-yl)-3-aryl- propan-1-one with 3-substituted-4-amino-5-mercapto-1,2,4-triazoles (Kalluraya *et al.*, 2002).

The asymmetric unit of the title compound consists of two crystallographically independent syndone molecules (B & C) (Fig. 1a), two crystallographically independent disordered syndone molecules (A & D) (Fig. 1b) and a water molecule with a refined site occupancy of 0.25. In the disordered syndone molecules, three atoms C1A, C2A, C3A  (molecule A) and C19D, C20D, C21D (molecule D) in the terminal phenyl rings are disordered over two positions, with occupancy ratios of 0.500 (18):0.500 (18) (molecule A) and 0.512 (17): 0.488 (17) (molecule D). The dihedral angles between terminal phenyl rings (C1A–C6A)/(C16A–C21A), (C1B–C6B)/(C16B–C21B), (C1C–C6C)/(C16C–C21C) and (C1D–C6D)/ (C16D–C21D) for all the syndone molecules are 23.3 (4)°, 45.57 (16)°, 68.46 (16)° and 56.5 (3)° respectively.

In the crystal structure (Fig. 2), all molecules are connected via intra and inter molecular N—H···N, N—H···O and C—H···O (Table 1) hydrogen bonds to form a three-dimensional network.

### Experimental

*N*-[(4-amino-5-mercapto-4*H*-1,2,4-triazol-3-yl)methyl]benzamide (0.01 mol) and 2,3-dibromo-1-(3'-phenylsydnon-4'-yl)-3-phenyl-propan-1-one (0.01 mol) in ethanol was treated with aqueous solution of sodium acetate (0.01 mol). The reaction mixture was refluxed on a water bath for 1–2 hours. The excess of ethanol was removed by distillation and the reaction mixture was kept overnight. The solid product separated was filtered.  It was then recrystallized from ethanol. Colourless needles were obtained from 1:2 mixtures of DMF and ethanol by slow evaporation.

### Refinement

Atoms H1NA, H2NA, H1NB, H2NB, H1NC, H2NC, H1ND and H2ND were located from a difference Fourier map and refined freely.  The remaining H atoms were positioned geometrically [N–H =0.78 (3)–0.89 (3)Å and C–H = 0.93–0.98 Å] and were refined using a riding model, with *U*_iso_(H) = 1.2 *U*_eq_(C, O). In the final refinement, the occupancies of the water molecules were fixed at 25%.  The C atoms C1A, C2A, C3A  (molecule A) and C19D, C20D, C21D (molecule D) of the terminal phenyl ring and the associated hydrogen atoms are disordered over two sites with a refined occupancy ratio of 0.500 (18): 0.500 (18) (molecule A) and 0.512 (17): 0.488 (17) (molecule D) respectively.

### Crystal data

**Table:**

C_27_H_21_N_7_O_4_S·0.06H_2_O	*Z* = 8
*M**_r_* = 540.69	*F*(000) = 2245
Triclinic, *P*1	*D*_x_ = 1.434 Mg m^−^^3^
Hall symbol: -P 1	Mo *K*α radiation, λ = 0.71073 Å
*a* = 15.6242 (19) Å	Cell parameters from 8028 reflections
*b* = 18.7430 (19) Å	θ = 2.7–26.3°
*c* = 18.9689 (19) Å	µ = 0.18 mm^−^^1^
α = 111.235 (2)°	*T* = 100 K
β = 93.970 (2)°	Needle, colourless
γ = 101.636 (2)°	0.64 × 0.08 × 0.07 mm
*V* = 5009.4 (9) Å^3^	

### Data collection

**Table:**

Bruker APEXII DUO CCD area-detector diffractometer	17604 independent reflections
Radiation source: fine-focus sealed tube	12624 reflections with *I* > 2σ(*I*)
graphite	*R*_int_ = 0.057
φ and ω scans	θ_max_ = 25.0°, θ_min_ = 2.0°
Absorption correction: multi-scan (*SADABS*; Bruker, 2009)	*h* = −18→18
*T*_min_ = 0.893, *T*_max_ = 0.988	*k* = −22→22
54193 measured reflections	*l* = −22→22

### Refinement

**Table:**

Refinement on *F*^2^	Primary atom site location: structure-invariant direct methods
Least-squares matrix: full	Secondary atom site location: difference Fourier map
*R*[*F*^2^ > 2σ(*F*^2^)] = 0.046	Hydrogen site location: inferred from neighbouring sites
*wR*(*F*^2^) = 0.141	H atoms treated by a mixture of independent and constrained refinement
*S* = 1.04	*w* = 1/[σ^2^(*F*_o_^2^) + (0.0647*P*)^2^ + 2.7459*P*] where *P* = (*F*_o_^2^ + 2*F*_c_^2^)/3
17604 reflections	(Δ/σ)_max_ = 0.001
1498 parameters	Δρ_max_ = 1.41 e Å^−^^3^
54 restraints	Δρ_min_ = −0.30 e Å^−^^3^

### Special details

**Table:**

Experimental. The crystal was placed in the cold stream of an Oxford Cryosystems Cobra open-flow nitrogen cryostat (Cosier & Glazer, 1986) operating at 100.0 (1) K.
Geometry. All s.u.'s (except the s.u. in the dihedral angle between two l.s. planes) are estimated using the full covariance matrix. The cell s.u.'s are taken into account individually in the estimation of s.u.'s in distances, angles and torsion angles; correlations between s.u.'s in cell parameters are only used when they are defined by crystal symmetry. An approximate (isotropic) treatment of cell s.u.'s is used for estimating s.u.'s involving l.s. planes.
Refinement. Refinement of F^2^ against ALL reflections. The weighted R-factor wR and goodness of fit S are based on F^2^, conventional R-factors R are based on F, with F set to zero for negative F^2^. The threshold expression of F^2^ > 2σ(F^2^) is used only for calculating R-factors(gt) etc. and is not relevant to the choice of reflections for refinement. R-factors based on F^2^ are statistically about twice as large as those based on F, and R- factors based on ALL data will be even larger.

### Fractional atomic coordinates and isotropic or equivalent isotropic displacement parameters (Å^2^)

**Table:**

	*x*	*y*	*z*	*U*_iso_*/*U*_eq_	Occ. (<1)
S1A	0.81870 (4)	0.17657 (4)	0.48519 (4)	0.02226 (16)	
O1A	0.63864 (15)	0.14942 (12)	0.67321 (12)	0.0361 (5)	
O2A	0.64762 (13)	0.12472 (12)	0.54756 (12)	0.0329 (5)	
O3A	0.92132 (14)	0.14012 (12)	0.63391 (11)	0.0307 (5)	
O4A	1.21342 (14)	0.03010 (12)	0.41242 (12)	0.0339 (5)	
N1A	0.76890 (18)	0.15234 (14)	0.71603 (13)	0.0310 (6)	
N2A	0.98017 (16)	0.09210 (14)	0.48506 (15)	0.0225 (5)	
N3A	0.6922 (2)	0.15895 (15)	0.73717 (15)	0.0392 (7)	
N4A	0.97880 (14)	0.14628 (13)	0.44937 (12)	0.0197 (5)	
N5A	0.93561 (15)	0.22744 (14)	0.40410 (13)	0.0250 (5)	
N6A	1.01463 (15)	0.21332 (14)	0.37903 (14)	0.0263 (5)	
N7A	1.17820 (16)	0.14717 (16)	0.45597 (17)	0.0349 (6)	
C1A	0.8463 (8)	0.2229 (5)	0.8387 (5)	0.035 (2)	0.500 (18)
H1AA	0.8164	0.2619	0.8422	0.042*	0.500 (18)
C2A	0.9055 (8)	0.2260 (6)	0.8986 (5)	0.046 (3)	0.500 (18)
H2AA	0.9199	0.2709	0.9436	0.055*	0.500 (18)
C3A	0.9429 (9)	0.1648 (11)	0.8929 (9)	0.048 (4)	0.500 (18)
H3AA	0.9836	0.1705	0.9340	0.058*	0.500 (18)
C1X	0.8951 (12)	0.2263 (5)	0.8283 (6)	0.054 (4)	0.500 (18)
H1XA	0.8903	0.2747	0.8278	0.064*	0.500 (18)
C2X	0.9591 (11)	0.2246 (6)	0.8821 (6)	0.058 (4)	0.500 (18)
H2XA	0.9925	0.2715	0.9199	0.069*	0.500 (18)
C3X	0.9725 (13)	0.1545 (10)	0.8794 (9)	0.048 (4)	0.500 (18)
H3XA	1.0153	0.1520	0.9144	0.057*	0.500 (18)
C4A	0.9222 (2)	0.0897 (2)	0.8249 (2)	0.0467 (9)	
H4AA	0.9476	0.0479	0.8212	0.056*	
C5A	0.8615 (2)	0.0879 (2)	0.76758 (18)	0.0355 (7)	
H5AA	0.8377	0.0413	0.7253	0.043*	
C6A	0.8365 (2)	0.15571 (19)	0.77376 (17)	0.0394 (8)	
C7A	0.6848 (2)	0.13492 (16)	0.60984 (18)	0.0280 (7)	
C8A	0.7708 (2)	0.13778 (16)	0.64035 (16)	0.0269 (6)	
C9A	0.84668 (19)	0.12511 (16)	0.60046 (16)	0.0237 (6)	
C10A	0.82464 (18)	0.09253 (16)	0.51319 (15)	0.0208 (6)	
H10A	0.7655	0.0568	0.4985	0.025*	
C11A	0.88942 (17)	0.04434 (15)	0.47488 (15)	0.0207 (6)	
H11A	0.8937	0.0088	0.5015	0.025*	
C12A	0.91619 (17)	0.18592 (15)	0.44596 (15)	0.0210 (6)	
C13A	1.03816 (17)	0.16366 (16)	0.40521 (16)	0.0237 (6)	
C14A	1.11474 (18)	0.12737 (18)	0.38790 (18)	0.0301 (7)	
H14A	1.0931	0.0705	0.3638	0.036*	
H14B	1.1444	0.1452	0.3518	0.036*	
C15A	1.22460 (17)	0.09663 (16)	0.46224 (17)	0.0236 (6)	
C16A	1.29184 (18)	0.12498 (16)	0.53308 (17)	0.0256 (6)	
C17A	1.2885 (2)	0.18610 (17)	0.60068 (18)	0.0299 (7)	
H17A	1.2435	0.2123	0.6034	0.036*	
C18A	1.3522 (2)	0.20800 (18)	0.66406 (19)	0.0343 (7)	
H18A	1.3496	0.2485	0.7095	0.041*	
C19A	1.4199 (2)	0.16944 (19)	0.65979 (19)	0.0353 (7)	
H19A	1.4630	0.1845	0.7023	0.042*	
C20A	1.4236 (2)	0.10878 (18)	0.59263 (18)	0.0327 (7)	
H20A	1.4695	0.0835	0.5897	0.039*	
C21A	1.35912 (18)	0.08568 (17)	0.52995 (17)	0.0268 (6)	
H21A	1.3606	0.0436	0.4853	0.032*	
C22A	0.85938 (18)	−0.00858 (16)	0.39070 (16)	0.0220 (6)	
C23A	0.91430 (19)	−0.05525 (16)	0.35411 (17)	0.0267 (6)	
H23A	0.9685	−0.0514	0.3806	0.032*	
C24A	0.8892 (2)	−0.10747 (18)	0.27856 (18)	0.0337 (7)	
H24A	0.9262	−0.1388	0.2547	0.040*	
C25A	0.8090 (2)	−0.11292 (18)	0.23861 (18)	0.0348 (7)	
H25A	0.7916	−0.1483	0.1880	0.042*	
C26A	0.7551 (2)	−0.06588 (17)	0.27391 (17)	0.0309 (7)	
H26A	0.7016	−0.0689	0.2468	0.037*	
C27A	0.77978 (18)	−0.01383 (17)	0.34973 (16)	0.0258 (6)	
H27A	0.7428	0.0177	0.3732	0.031*	
S1B	0.02213 (5)	0.33764 (4)	0.63624 (4)	0.02532 (17)	
O1B	−0.24522 (14)	0.32349 (12)	0.73433 (12)	0.0325 (5)	
O2B	−0.09434 (15)	0.36214 (13)	0.75883 (12)	0.0374 (5)	
O3B	−0.14885 (12)	0.37946 (12)	0.53670 (11)	0.0285 (5)	
O4B	0.13564 (14)	0.45708 (11)	0.31579 (12)	0.0340 (5)	
N1B	−0.27213 (15)	0.34360 (13)	0.63281 (13)	0.0253 (5)	
N2B	−0.31183 (17)	0.32007 (14)	0.68140 (15)	0.0323 (6)	
N3B	0.03196 (16)	0.42385 (14)	0.51785 (13)	0.0219 (5)	
N4B	0.08836 (14)	0.37506 (13)	0.52056 (12)	0.0206 (5)	
N5B	0.16085 (16)	0.30654 (14)	0.56221 (14)	0.0298 (6)	
N6B	0.20166 (16)	0.32278 (15)	0.50383 (14)	0.0316 (6)	
N7B	0.10437 (16)	0.35620 (15)	0.35535 (13)	0.0251 (5)	
C1B	−0.3217 (2)	0.42780 (17)	0.57623 (17)	0.0290 (7)	
H1BA	−0.2784	0.4704	0.6107	0.035*	
C2B	−0.3811 (2)	0.43785 (18)	0.52553 (18)	0.0332 (7)	
H2BA	−0.3780	0.4879	0.5254	0.040*	
C3B	−0.4453 (2)	0.3741 (2)	0.4750 (2)	0.0406 (8)	
H3BA	−0.4859	0.3816	0.4417	0.049*	
C4B	−0.4493 (2)	0.2992 (2)	0.4735 (2)	0.0490 (9)	
H4BA	−0.4914	0.2562	0.4381	0.059*	
C5B	−0.3909 (2)	0.28803 (18)	0.5246 (2)	0.0406 (8)	
H5BA	−0.3940	0.2382	0.5252	0.049*	
C6B	−0.32852 (18)	0.35245 (17)	0.57401 (17)	0.0270 (6)	
C7B	−0.1614 (2)	0.35327 (17)	0.71769 (17)	0.0288 (7)	
C8B	−0.18205 (18)	0.36415 (16)	0.64914 (16)	0.0238 (6)	
C9B	−0.12279 (18)	0.38757 (16)	0.60165 (16)	0.0230 (6)	
C10B	−0.02561 (18)	0.42104 (16)	0.63779 (16)	0.0222 (6)	
H10B	−0.0231	0.4553	0.6914	0.027*	
C11B	0.02394 (18)	0.47146 (16)	0.59823 (15)	0.0217 (6)	
H11B	−0.0119	0.5079	0.5955	0.026*	
C12B	0.09345 (18)	0.33827 (15)	0.57003 (15)	0.0223 (6)	
C13B	0.15782 (18)	0.36470 (17)	0.48147 (16)	0.0257 (6)	
C14B	0.17561 (19)	0.39461 (18)	0.42032 (16)	0.0301 (7)	
H14C	0.1814	0.4512	0.4403	0.036*	
H14D	0.2309	0.3848	0.4037	0.036*	
C15B	0.08921 (19)	0.39222 (16)	0.30756 (16)	0.0259 (6)	
C16B	0.01206 (19)	0.35136 (16)	0.24481 (16)	0.0253 (6)	
C17B	−0.0610 (2)	0.29786 (17)	0.24864 (17)	0.0295 (7)	
H17B	−0.0619	0.2831	0.2905	0.035*	
C18B	−0.1326 (2)	0.26633 (19)	0.19035 (19)	0.0397 (8)	
H18B	−0.1819	0.2308	0.1932	0.048*	
C19B	−0.1306 (3)	0.2876 (2)	0.1282 (2)	0.0487 (9)	
H19B	−0.1788	0.2660	0.0890	0.058*	
C20B	−0.0585 (3)	0.3401 (2)	0.1232 (2)	0.0514 (10)	
H20B	−0.0577	0.3537	0.0807	0.062*	
C21B	0.0128 (2)	0.3726 (2)	0.18164 (19)	0.0395 (8)	
H21B	0.0614	0.4088	0.1788	0.047*	
C22B	0.11491 (18)	0.52202 (15)	0.64050 (16)	0.0220 (6)	
C23B	0.16575 (19)	0.56588 (17)	0.60627 (17)	0.0287 (7)	
H23B	0.1433	0.5636	0.5586	0.034*	
C24B	0.2489 (2)	0.61289 (18)	0.64159 (18)	0.0336 (7)	
H24B	0.2822	0.6418	0.6177	0.040*	
C25B	0.2827 (2)	0.61692 (18)	0.71262 (19)	0.0351 (7)	
H25B	0.3386	0.6488	0.7368	0.042*	
C26B	0.2334 (2)	0.57374 (18)	0.74723 (19)	0.0348 (7)	
H26B	0.2560	0.5764	0.7950	0.042*	
C27B	0.15042 (19)	0.52630 (16)	0.71131 (16)	0.0275 (7)	
H27B	0.1179	0.4968	0.7350	0.033*	
S1C	0.48300 (5)	0.26223 (4)	0.15697 (4)	0.02559 (17)	
O1C	0.19815 (15)	0.16423 (15)	0.22509 (14)	0.0466 (6)	
O2C	0.34340 (16)	0.22827 (14)	0.26351 (13)	0.0414 (6)	
O3C	0.34333 (12)	0.08877 (12)	0.02058 (12)	0.0299 (5)	
O4C	0.69901 (14)	0.00973 (13)	−0.13738 (13)	0.0399 (6)	
N1C	0.19460 (16)	0.10302 (15)	0.10598 (16)	0.0328 (6)	
N2C	0.14369 (18)	0.11339 (19)	0.15804 (18)	0.0462 (7)	
N3C	0.53334 (16)	0.11028 (13)	0.03307 (14)	0.0228 (5)	
N4C	0.57434 (14)	0.18924 (13)	0.04532 (13)	0.0207 (5)	
N5C	0.59980 (17)	0.31759 (14)	0.07845 (14)	0.0304 (6)	
N6C	0.65470 (16)	0.28738 (15)	0.02506 (14)	0.0304 (6)	
N7C	0.62830 (16)	0.10687 (15)	−0.11771 (14)	0.0262 (6)	
C1C	0.1262 (2)	−0.0297 (2)	0.0143 (2)	0.0429 (9)	
H1CA	0.1349	−0.0485	0.0528	0.051*	
C2C	0.0858 (2)	−0.0807 (2)	−0.0585 (2)	0.0478 (9)	
H2CA	0.0662	−0.1343	−0.0692	0.057*	
C3C	0.0743 (2)	−0.0528 (2)	−0.1155 (2)	0.0487 (9)	
H3CA	0.0473	−0.0875	−0.1646	0.058*	
C4C	0.1029 (2)	0.0270 (3)	−0.0995 (2)	0.0519 (10)	
H4CA	0.0956	0.0457	−0.1382	0.062*	
C5C	0.1423 (2)	0.0793 (2)	−0.0264 (2)	0.0448 (9)	
H5CA	0.1608	0.1332	−0.0150	0.054*	
C6C	0.15331 (19)	0.04922 (19)	0.02876 (19)	0.0338 (7)	
C7C	0.2858 (2)	0.18375 (19)	0.2114 (2)	0.0349 (8)	
C8C	0.28101 (19)	0.14226 (17)	0.13221 (18)	0.0286 (7)	
C9C	0.35350 (18)	0.12604 (16)	0.08981 (18)	0.0256 (6)	
C10C	0.44429 (18)	0.15702 (16)	0.13905 (16)	0.0239 (6)	
H10C	0.4362	0.1536	0.1886	0.029*	
C11C	0.50822 (17)	0.10585 (16)	0.10525 (15)	0.0214 (6)	
H11C	0.4746	0.0513	0.0913	0.026*	
C12C	0.55301 (18)	0.25668 (16)	0.08875 (16)	0.0239 (6)	
C13C	0.63826 (18)	0.21076 (17)	0.00678 (15)	0.0255 (6)	
C14C	0.68399 (19)	0.15572 (18)	−0.04461 (16)	0.0295 (7)	
H14E	0.7377	0.1857	−0.0532	0.035*	
H14F	0.7007	0.1221	−0.0201	0.035*	
C15C	0.64339 (18)	0.03716 (17)	−0.16049 (17)	0.0258 (6)	
C16C	0.58866 (18)	−0.00515 (16)	−0.23813 (16)	0.0245 (6)	
C17C	0.5075 (2)	0.00949 (17)	−0.25619 (18)	0.0312 (7)	
H17C	0.4838	0.0447	−0.2191	0.037*	
C18C	0.4630 (2)	−0.02942 (19)	−0.33027 (19)	0.0389 (8)	
H18C	0.4088	−0.0205	−0.3432	0.047*	
C19C	0.4982 (2)	−0.08132 (19)	−0.38510 (19)	0.0417 (9)	
H19C	0.4682	−0.1060	−0.4351	0.050*	
C20C	0.5774 (2)	−0.09711 (19)	−0.36676 (19)	0.0402 (8)	
H20C	0.6003	−0.1331	−0.4038	0.048*	
C21C	0.6220 (2)	−0.05916 (17)	−0.29329 (17)	0.0320 (7)	
H21C	0.6752	−0.0699	−0.2804	0.038*	
C22C	0.58901 (18)	0.11634 (16)	0.16028 (16)	0.0242 (6)	
C23C	0.6062 (2)	0.16828 (17)	0.23681 (18)	0.0322 (7)	
H23C	0.5692	0.2016	0.2556	0.039*	
C24C	0.6789 (2)	0.17039 (19)	0.2853 (2)	0.0406 (8)	
H24C	0.6902	0.2052	0.3363	0.049*	
C25C	0.7340 (2)	0.1210 (2)	0.2579 (2)	0.0427 (9)	
H25C	0.7824	0.1225	0.2904	0.051*	
C26C	0.7171 (2)	0.0696 (2)	0.1825 (2)	0.0388 (8)	
H26C	0.7539	0.0359	0.1640	0.047*	
C27C	0.64560 (19)	0.06778 (18)	0.13399 (19)	0.0309 (7)	
H27C	0.6354	0.0334	0.0829	0.037*	
S1D	0.67540 (5)	0.74950 (4)	0.01406 (4)	0.02542 (17)	
O1D	0.77315 (16)	0.67127 (13)	−0.22023 (13)	0.0414 (6)	
O2D	0.81168 (15)	0.71952 (13)	−0.09094 (14)	0.0377 (5)	
O3D	0.54384 (14)	0.56927 (13)	−0.12470 (11)	0.0338 (5)	
O4D	0.30364 (16)	0.53490 (12)	0.13597 (12)	0.0395 (5)	
N1D	0.64817 (17)	0.59195 (14)	−0.23674 (14)	0.0303 (6)	
N2D	0.7045 (2)	0.61867 (17)	−0.27436 (16)	0.0407 (7)	
N3D	0.52802 (16)	0.61112 (13)	0.03269 (14)	0.0252 (5)	
N4D	0.53310 (15)	0.69244 (13)	0.07243 (12)	0.0222 (5)	
N5D	0.56679 (15)	0.82093 (14)	0.10592 (14)	0.0284 (6)	
N6D	0.49628 (15)	0.79944 (15)	0.14236 (14)	0.0301 (6)	
N7D	0.33468 (15)	0.62925 (14)	0.08854 (14)	0.0259 (5)	
C1D	0.5087 (3)	0.5518 (2)	−0.32158 (18)	0.0445 (9)	
H1DA	0.5157	0.6033	−0.3187	0.053*	
C2D	0.4363 (3)	0.4928 (3)	−0.3663 (2)	0.0539 (11)	
H2DA	0.3932	0.5049	−0.3933	0.065*	
C3D	0.4268 (2)	0.4159 (2)	−0.37144 (19)	0.0448 (9)	
H3DA	0.3781	0.3766	−0.4024	0.054*	
C4D	0.4899 (2)	0.3975 (2)	−0.33049 (18)	0.0375 (8)	
H4DA	0.4838	0.3458	−0.3342	0.045*	
C5D	0.5613 (2)	0.45567 (18)	−0.28440 (17)	0.0317 (7)	
H5DA	0.6033	0.4443	−0.2556	0.038*	
C6D	0.5697 (2)	0.53196 (18)	−0.28169 (16)	0.0316 (7)	
C7D	0.7583 (2)	0.67542 (19)	−0.14649 (19)	0.0313 (7)	
C8D	0.6751 (2)	0.62235 (17)	−0.16016 (16)	0.0274 (7)	
C9D	0.6211 (2)	0.60676 (17)	−0.10561 (17)	0.0272 (7)	
C10D	0.66614 (19)	0.64259 (16)	−0.02236 (15)	0.0243 (6)	
H10D	0.7263	0.6348	−0.0219	0.029*	
C11D	0.61791 (19)	0.59872 (16)	0.02358 (16)	0.0243 (6)	
H11D	0.6094	0.5424	−0.0074	0.029*	
C12D	0.58650 (18)	0.75537 (16)	0.06481 (16)	0.0232 (6)	
C13D	0.47891 (18)	0.72224 (17)	0.12128 (16)	0.0261 (6)	
C14D	0.41083 (19)	0.6744 (2)	0.14785 (17)	0.0326 (7)	
H14G	0.4368	0.6385	0.1634	0.039*	
H14H	0.3915	0.7092	0.1922	0.039*	
C15D	0.2857 (2)	0.56152 (17)	0.08784 (16)	0.0273 (6)	
C16D	0.2073 (2)	0.52059 (17)	0.02742 (17)	0.0298 (7)	
C17D	0.1970 (2)	0.53132 (18)	−0.04070 (17)	0.0309 (7)	
H17D	0.2406	0.5673	−0.0499	0.037*	
C18D	0.1229 (2)	0.4895 (2)	−0.09543 (19)	0.0451 (9)	
H18D	0.1255	0.4871	−0.1450	0.054*	
C19D	0.0480 (8)	0.4523 (7)	−0.0815 (6)	0.043 (2)	0.512 (17)
H19D	−0.0035	0.4319	−0.1172	0.051*	0.512 (17)
C20D	0.0509 (7)	0.4456 (6)	−0.0113 (5)	0.048 (3)	0.512 (17)
H20D	0.0010	0.4185	0.0005	0.058*	0.512 (17)
C21D	0.1282 (8)	0.4792 (7)	0.0418 (5)	0.040 (3)	0.512 (17)
H21D	0.1281	0.4743	0.0889	0.048*	0.512 (17)
C19X	0.0692 (7)	0.4219 (9)	−0.0848 (6)	0.044 (3)	0.488 (17)
H19E	0.0223	0.3883	−0.1227	0.053*	0.488 (17)
C20X	0.0857 (6)	0.4063 (7)	−0.0207 (5)	0.041 (3)	0.488 (17)
H20E	0.0484	0.3639	−0.0150	0.050*	0.488 (17)
C21X	0.1575 (7)	0.4531 (6)	0.0360 (5)	0.031 (2)	0.488 (17)
H21E	0.1720	0.4406	0.0778	0.037*	0.488 (17)
C22D	0.6686 (2)	0.61265 (16)	0.10021 (16)	0.0270 (6)	
C23D	0.7508 (2)	0.66351 (18)	0.13177 (18)	0.0368 (8)	
H23D	0.7777	0.6941	0.1065	0.044*	
C24D	0.7940 (3)	0.6694 (2)	0.20131 (19)	0.0455 (9)	
H24D	0.8493	0.7039	0.2224	0.055*	
C25D	0.7542 (3)	0.6238 (2)	0.23845 (18)	0.0434 (9)	
H25D	0.7829	0.6275	0.2847	0.052*	
C26D	0.6728 (2)	0.5730 (2)	0.20766 (18)	0.0396 (8)	
H26D	0.6465	0.5419	0.2327	0.048*	
C27D	0.6299 (2)	0.56810 (18)	0.13995 (17)	0.0318 (7)	
H27D	0.5739	0.5344	0.1201	0.038*	
H1NA	1.193 (3)	0.200 (3)	0.490 (2)	0.065 (12)*	
H2NA	0.998 (2)	0.1156 (18)	0.5284 (18)	0.023 (9)*	
H1NB	0.0761 (19)	0.3090 (19)	0.3471 (17)	0.025 (8)*	
H2NB	−0.018 (2)	0.3892 (18)	0.4936 (18)	0.028 (9)*	
H1NC	0.595 (2)	0.1273 (19)	−0.1330 (18)	0.029 (9)*	
H2NC	0.482 (2)	0.0965 (17)	0.0007 (17)	0.028 (8)*	
H1ND	0.3206 (19)	0.6484 (17)	0.0539 (17)	0.027 (8)*	
H2ND	0.502 (2)	0.593 (2)	−0.019 (2)	0.046 (10)*	
O1W	0.3466 (6)	0.2769 (5)	0.4204 (5)	0.042 (2)*	0.25
H1W1	0.3021	0.2878	0.4418	0.064*	0.25
H2W1	0.3486	0.2631	0.3727	0.064*	0.25

### Atomic displacement parameters (Å^2^)

**Table:**

	*U*^11^	*U*^22^	*U*^33^	*U*^12^	*U*^13^	*U*^23^
S1A	0.0232 (4)	0.0242 (4)	0.0232 (4)	0.0071 (3)	0.0038 (3)	0.0127 (3)
O1A	0.0481 (13)	0.0325 (12)	0.0348 (13)	0.0153 (10)	0.0233 (11)	0.0151 (10)
O2A	0.0354 (12)	0.0379 (12)	0.0329 (13)	0.0173 (10)	0.0087 (10)	0.0171 (10)
O3A	0.0362 (12)	0.0353 (12)	0.0205 (11)	0.0102 (10)	−0.0004 (9)	0.0107 (9)
O4A	0.0463 (13)	0.0289 (12)	0.0271 (12)	0.0157 (10)	0.0048 (10)	0.0081 (10)
N1A	0.0559 (17)	0.0210 (13)	0.0194 (13)	0.0106 (12)	0.0119 (12)	0.0097 (11)
N2A	0.0248 (13)	0.0236 (13)	0.0206 (14)	0.0049 (10)	−0.0016 (11)	0.0117 (12)
N3A	0.0622 (19)	0.0297 (14)	0.0314 (16)	0.0118 (14)	0.0225 (15)	0.0152 (13)
N4A	0.0206 (12)	0.0213 (12)	0.0179 (12)	0.0024 (9)	−0.0003 (9)	0.0103 (10)
N5A	0.0223 (12)	0.0290 (13)	0.0265 (13)	0.0028 (10)	0.0023 (10)	0.0160 (11)
N6A	0.0218 (12)	0.0291 (13)	0.0308 (14)	0.0046 (10)	0.0037 (10)	0.0158 (11)
N7A	0.0271 (14)	0.0231 (14)	0.0468 (17)	0.0044 (11)	−0.0104 (12)	0.0085 (13)
C1A	0.034 (5)	0.045 (4)	0.019 (4)	−0.007 (4)	0.004 (4)	0.015 (3)
C2A	0.044 (6)	0.055 (5)	0.025 (4)	−0.015 (4)	0.001 (4)	0.016 (4)
C3A	0.037 (7)	0.079 (10)	0.020 (6)	−0.011 (6)	−0.002 (4)	0.025 (6)
C1X	0.085 (9)	0.025 (4)	0.043 (5)	0.012 (5)	−0.019 (6)	0.010 (4)
C2X	0.083 (10)	0.042 (5)	0.034 (5)	0.014 (5)	−0.025 (5)	0.005 (4)
C3X	0.081 (11)	0.048 (5)	0.027 (6)	0.033 (7)	0.012 (6)	0.018 (4)
C4A	0.046 (2)	0.062 (2)	0.038 (2)	0.0108 (19)	0.0044 (17)	0.026 (2)
C5A	0.0353 (18)	0.0410 (19)	0.0288 (18)	0.0095 (15)	0.0059 (14)	0.0115 (15)
C6A	0.069 (2)	0.0311 (17)	0.0152 (16)	0.0019 (16)	0.0015 (15)	0.0113 (14)
C7A	0.0390 (18)	0.0211 (15)	0.0300 (18)	0.0115 (13)	0.0179 (15)	0.0125 (13)
C8A	0.0425 (18)	0.0233 (15)	0.0196 (15)	0.0131 (13)	0.0080 (13)	0.0105 (13)
C9A	0.0349 (17)	0.0204 (14)	0.0195 (15)	0.0095 (12)	0.0044 (13)	0.0105 (12)
C10A	0.0231 (14)	0.0222 (14)	0.0190 (15)	0.0037 (11)	0.0008 (11)	0.0114 (12)
C11A	0.0242 (14)	0.0215 (14)	0.0200 (15)	0.0063 (12)	0.0036 (12)	0.0118 (12)
C12A	0.0196 (14)	0.0209 (14)	0.0207 (15)	0.0012 (11)	−0.0036 (11)	0.0092 (12)
C13A	0.0204 (14)	0.0253 (15)	0.0250 (16)	0.0004 (12)	0.0010 (12)	0.0125 (13)
C14A	0.0224 (15)	0.0356 (17)	0.0346 (18)	0.0053 (13)	0.0029 (13)	0.0175 (15)
C15A	0.0208 (14)	0.0228 (15)	0.0325 (17)	0.0035 (12)	0.0106 (12)	0.0164 (14)
C16A	0.0237 (15)	0.0236 (15)	0.0339 (17)	0.0030 (12)	0.0070 (13)	0.0170 (14)
C17A	0.0317 (17)	0.0239 (15)	0.0370 (18)	0.0107 (13)	0.0083 (14)	0.0126 (14)
C18A	0.0382 (18)	0.0288 (16)	0.0336 (18)	0.0092 (14)	0.0052 (15)	0.0090 (14)
C19A	0.0321 (17)	0.0381 (18)	0.0341 (19)	0.0048 (14)	−0.0015 (14)	0.0151 (16)
C20A	0.0279 (16)	0.0368 (18)	0.042 (2)	0.0139 (14)	0.0096 (14)	0.0212 (16)
C21A	0.0295 (16)	0.0246 (15)	0.0306 (17)	0.0097 (13)	0.0090 (13)	0.0132 (13)
C22A	0.0258 (15)	0.0211 (14)	0.0225 (15)	0.0030 (12)	0.0053 (12)	0.0135 (12)
C23A	0.0280 (16)	0.0261 (15)	0.0286 (17)	0.0061 (13)	0.0068 (13)	0.0132 (14)
C24A	0.0403 (19)	0.0286 (16)	0.0324 (18)	0.0098 (14)	0.0133 (15)	0.0098 (14)
C25A	0.049 (2)	0.0259 (16)	0.0210 (16)	−0.0005 (15)	0.0052 (15)	0.0043 (13)
C26A	0.0360 (17)	0.0285 (16)	0.0245 (17)	0.0042 (14)	−0.0007 (14)	0.0088 (14)
C27A	0.0266 (15)	0.0269 (15)	0.0240 (16)	0.0045 (12)	0.0040 (12)	0.0110 (13)
S1B	0.0342 (4)	0.0249 (4)	0.0228 (4)	0.0088 (3)	0.0059 (3)	0.0148 (3)
O1B	0.0407 (12)	0.0306 (11)	0.0305 (12)	0.0062 (10)	0.0145 (10)	0.0165 (10)
O2B	0.0431 (14)	0.0463 (14)	0.0266 (12)	0.0044 (11)	0.0050 (11)	0.0218 (11)
O3B	0.0283 (11)	0.0378 (12)	0.0261 (12)	0.0095 (9)	0.0083 (9)	0.0186 (10)
O4B	0.0404 (12)	0.0198 (10)	0.0436 (14)	0.0049 (9)	0.0132 (10)	0.0142 (10)
N1B	0.0321 (14)	0.0201 (12)	0.0280 (14)	0.0078 (10)	0.0154 (11)	0.0116 (11)
N2B	0.0369 (15)	0.0277 (14)	0.0377 (16)	0.0072 (11)	0.0145 (13)	0.0177 (12)
N3B	0.0232 (13)	0.0270 (13)	0.0196 (13)	0.0102 (11)	0.0043 (10)	0.0113 (11)
N4B	0.0216 (12)	0.0234 (12)	0.0163 (12)	0.0067 (10)	0.0020 (10)	0.0066 (10)
N5B	0.0354 (14)	0.0285 (13)	0.0236 (14)	0.0139 (11)	−0.0007 (11)	0.0055 (11)
N6B	0.0324 (14)	0.0346 (14)	0.0218 (14)	0.0123 (12)	0.0032 (11)	0.0022 (12)
N7B	0.0312 (14)	0.0211 (13)	0.0197 (13)	−0.0010 (11)	0.0062 (11)	0.0078 (11)
C1B	0.0339 (17)	0.0249 (15)	0.0314 (17)	0.0074 (13)	0.0160 (14)	0.0126 (14)
C2B	0.0417 (19)	0.0280 (16)	0.0380 (19)	0.0128 (15)	0.0171 (15)	0.0177 (15)
C3B	0.0360 (19)	0.044 (2)	0.051 (2)	0.0141 (16)	0.0085 (16)	0.0250 (18)
C4B	0.041 (2)	0.0337 (19)	0.065 (3)	0.0020 (16)	−0.0092 (18)	0.0177 (19)
C5B	0.0408 (19)	0.0210 (16)	0.057 (2)	0.0048 (14)	0.0005 (17)	0.0149 (16)
C6B	0.0258 (15)	0.0257 (15)	0.0327 (17)	0.0095 (13)	0.0122 (13)	0.0119 (14)
C7B	0.0408 (19)	0.0226 (15)	0.0263 (17)	0.0081 (13)	0.0170 (15)	0.0111 (13)
C8B	0.0295 (16)	0.0193 (14)	0.0230 (15)	0.0052 (12)	0.0090 (12)	0.0082 (12)
C9B	0.0296 (16)	0.0204 (14)	0.0221 (16)	0.0097 (12)	0.0096 (12)	0.0088 (12)
C10B	0.0285 (15)	0.0212 (14)	0.0189 (15)	0.0070 (12)	0.0053 (12)	0.0092 (12)
C11B	0.0263 (15)	0.0247 (14)	0.0213 (15)	0.0108 (12)	0.0092 (12)	0.0139 (12)
C12B	0.0312 (16)	0.0178 (13)	0.0140 (14)	0.0049 (12)	−0.0014 (12)	0.0031 (11)
C13B	0.0223 (15)	0.0303 (16)	0.0191 (15)	0.0051 (13)	0.0024 (12)	0.0043 (13)
C14B	0.0266 (16)	0.0344 (17)	0.0227 (16)	−0.0001 (13)	0.0050 (13)	0.0072 (14)
C15B	0.0346 (17)	0.0223 (15)	0.0235 (16)	0.0104 (13)	0.0173 (13)	0.0078 (13)
C16B	0.0366 (17)	0.0222 (14)	0.0217 (15)	0.0137 (13)	0.0112 (13)	0.0089 (12)
C17B	0.0415 (18)	0.0238 (15)	0.0240 (16)	0.0093 (14)	0.0023 (14)	0.0098 (13)
C18B	0.048 (2)	0.0310 (17)	0.0350 (19)	0.0075 (15)	−0.0061 (16)	0.0095 (15)
C19B	0.059 (2)	0.050 (2)	0.031 (2)	0.0192 (19)	−0.0102 (17)	0.0089 (17)
C20B	0.071 (3)	0.071 (3)	0.030 (2)	0.037 (2)	0.0141 (19)	0.0289 (19)
C21B	0.050 (2)	0.048 (2)	0.0355 (19)	0.0218 (17)	0.0181 (17)	0.0265 (17)
C22B	0.0289 (15)	0.0191 (14)	0.0217 (15)	0.0113 (12)	0.0086 (12)	0.0083 (12)
C23B	0.0350 (17)	0.0269 (15)	0.0278 (17)	0.0077 (13)	0.0099 (14)	0.0139 (14)
C24B	0.0363 (18)	0.0268 (16)	0.0373 (19)	0.0050 (14)	0.0129 (15)	0.0122 (15)
C25B	0.0317 (17)	0.0275 (16)	0.0375 (19)	0.0028 (14)	0.0011 (14)	0.0058 (15)
C26B	0.0394 (19)	0.0308 (17)	0.0306 (18)	0.0049 (14)	−0.0015 (14)	0.0109 (15)
C27B	0.0357 (17)	0.0225 (15)	0.0240 (16)	0.0056 (13)	0.0064 (13)	0.0091 (13)
S1C	0.0299 (4)	0.0212 (4)	0.0263 (4)	0.0079 (3)	0.0059 (3)	0.0086 (3)
O1C	0.0396 (14)	0.0583 (16)	0.0510 (16)	0.0172 (12)	0.0252 (12)	0.0256 (13)
O2C	0.0451 (14)	0.0393 (13)	0.0391 (14)	0.0163 (12)	0.0177 (12)	0.0091 (12)
O3C	0.0246 (11)	0.0335 (12)	0.0291 (13)	0.0042 (9)	0.0037 (9)	0.0109 (10)
O4C	0.0427 (13)	0.0402 (13)	0.0395 (13)	0.0149 (11)	−0.0074 (11)	0.0181 (11)
N1C	0.0264 (14)	0.0393 (15)	0.0457 (17)	0.0119 (12)	0.0173 (13)	0.0270 (14)
N2C	0.0346 (16)	0.062 (2)	0.052 (2)	0.0147 (15)	0.0224 (15)	0.0296 (17)
N3C	0.0225 (13)	0.0217 (12)	0.0211 (13)	0.0018 (10)	0.0028 (11)	0.0068 (10)
N4C	0.0220 (12)	0.0195 (12)	0.0194 (12)	0.0022 (10)	0.0015 (10)	0.0080 (10)
N5C	0.0399 (15)	0.0253 (13)	0.0245 (14)	0.0003 (11)	0.0002 (12)	0.0127 (11)
N6C	0.0360 (14)	0.0316 (14)	0.0202 (13)	−0.0018 (11)	0.0024 (11)	0.0117 (11)
N7C	0.0279 (14)	0.0342 (15)	0.0191 (13)	0.0096 (12)	0.0036 (11)	0.0122 (12)
C1C	0.040 (2)	0.049 (2)	0.053 (2)	0.0169 (17)	0.0129 (17)	0.0319 (19)
C2C	0.042 (2)	0.049 (2)	0.053 (2)	0.0106 (17)	0.0084 (18)	0.020 (2)
C3C	0.0249 (18)	0.074 (3)	0.047 (2)	0.0086 (18)	0.0055 (16)	0.025 (2)
C4C	0.0234 (18)	0.087 (3)	0.059 (3)	0.0050 (19)	0.0008 (17)	0.049 (2)
C5C	0.0239 (17)	0.059 (2)	0.064 (3)	0.0033 (16)	0.0026 (17)	0.042 (2)
C6C	0.0179 (15)	0.0454 (19)	0.047 (2)	0.0082 (14)	0.0102 (14)	0.0268 (17)
C7C	0.0368 (19)	0.0317 (17)	0.047 (2)	0.0162 (15)	0.0199 (17)	0.0207 (17)
C8C	0.0267 (16)	0.0289 (16)	0.0382 (19)	0.0096 (13)	0.0121 (14)	0.0194 (15)
C9C	0.0255 (15)	0.0231 (15)	0.0338 (19)	0.0065 (12)	0.0075 (13)	0.0166 (14)
C10C	0.0276 (15)	0.0214 (14)	0.0253 (16)	0.0051 (12)	0.0075 (12)	0.0117 (13)
C11C	0.0238 (14)	0.0187 (14)	0.0228 (15)	0.0049 (11)	0.0075 (12)	0.0086 (12)
C12C	0.0278 (15)	0.0249 (15)	0.0172 (15)	0.0016 (12)	−0.0038 (12)	0.0101 (12)
C13C	0.0234 (15)	0.0332 (17)	0.0156 (15)	−0.0042 (12)	−0.0029 (12)	0.0114 (13)
C14C	0.0265 (16)	0.0397 (18)	0.0186 (15)	0.0037 (13)	0.0040 (12)	0.0091 (14)
C15C	0.0277 (16)	0.0271 (15)	0.0268 (16)	0.0033 (13)	0.0045 (13)	0.0170 (14)
C16C	0.0273 (15)	0.0229 (14)	0.0252 (16)	0.0017 (12)	0.0039 (12)	0.0138 (13)
C17C	0.0327 (17)	0.0239 (15)	0.0347 (18)	0.0037 (13)	−0.0020 (14)	0.0118 (14)
C18C	0.0378 (19)	0.0325 (18)	0.041 (2)	0.0010 (15)	−0.0139 (16)	0.0155 (16)
C19C	0.054 (2)	0.0313 (18)	0.0261 (18)	−0.0091 (16)	−0.0094 (16)	0.0083 (15)
C20C	0.049 (2)	0.0311 (18)	0.0320 (19)	−0.0014 (16)	0.0083 (16)	0.0082 (15)
C21C	0.0352 (17)	0.0288 (16)	0.0310 (18)	0.0033 (14)	0.0069 (14)	0.0124 (14)
C22C	0.0241 (15)	0.0224 (14)	0.0281 (16)	0.0029 (12)	0.0056 (12)	0.0132 (13)
C23C	0.0422 (18)	0.0231 (15)	0.0326 (18)	0.0098 (14)	−0.0008 (14)	0.0126 (14)
C24C	0.054 (2)	0.0298 (17)	0.0330 (19)	0.0019 (16)	−0.0104 (16)	0.0143 (15)
C25C	0.0368 (19)	0.042 (2)	0.051 (2)	0.0013 (16)	−0.0110 (17)	0.0285 (19)
C26C	0.0275 (17)	0.0404 (19)	0.056 (2)	0.0128 (15)	0.0066 (16)	0.0254 (18)
C27C	0.0272 (16)	0.0325 (17)	0.0349 (18)	0.0066 (13)	0.0050 (14)	0.0156 (15)
S1D	0.0302 (4)	0.0243 (4)	0.0256 (4)	0.0069 (3)	0.0088 (3)	0.0131 (3)
O1D	0.0602 (16)	0.0350 (13)	0.0376 (14)	0.0171 (12)	0.0295 (12)	0.0172 (11)
O2D	0.0384 (13)	0.0338 (12)	0.0449 (15)	0.0114 (11)	0.0188 (11)	0.0160 (12)
O3D	0.0349 (13)	0.0419 (13)	0.0197 (11)	0.0028 (10)	0.0059 (9)	0.0094 (10)
O4D	0.0606 (15)	0.0336 (12)	0.0302 (12)	0.0125 (11)	−0.0001 (11)	0.0197 (11)
N1D	0.0503 (16)	0.0297 (14)	0.0236 (14)	0.0234 (13)	0.0186 (12)	0.0154 (12)
N2D	0.0631 (19)	0.0424 (16)	0.0319 (16)	0.0243 (15)	0.0277 (15)	0.0222 (14)
N3D	0.0313 (14)	0.0221 (12)	0.0176 (13)	0.0004 (10)	0.0039 (11)	0.0055 (11)
N4D	0.0271 (13)	0.0218 (12)	0.0154 (12)	0.0030 (10)	0.0028 (10)	0.0061 (10)
N5D	0.0266 (13)	0.0283 (13)	0.0283 (14)	0.0061 (11)	0.0038 (11)	0.0090 (12)
N6D	0.0253 (13)	0.0332 (14)	0.0274 (14)	0.0066 (11)	0.0047 (11)	0.0069 (12)
N7D	0.0260 (13)	0.0305 (14)	0.0227 (14)	0.0025 (11)	0.0018 (11)	0.0149 (12)
C1D	0.073 (3)	0.049 (2)	0.0204 (17)	0.037 (2)	0.0065 (17)	0.0115 (16)
C2D	0.069 (3)	0.083 (3)	0.0243 (19)	0.050 (2)	0.0074 (18)	0.020 (2)
C3D	0.046 (2)	0.064 (2)	0.0220 (17)	0.0215 (19)	0.0064 (15)	0.0102 (17)
C4D	0.0425 (19)	0.047 (2)	0.0269 (18)	0.0125 (16)	0.0141 (15)	0.0164 (16)
C5D	0.0411 (18)	0.0405 (18)	0.0242 (16)	0.0179 (15)	0.0128 (14)	0.0192 (15)
C6D	0.050 (2)	0.0385 (18)	0.0151 (15)	0.0247 (16)	0.0131 (14)	0.0115 (14)
C7D	0.0434 (19)	0.0307 (17)	0.0337 (19)	0.0204 (15)	0.0246 (16)	0.0190 (15)
C8D	0.0401 (18)	0.0282 (16)	0.0226 (16)	0.0165 (14)	0.0135 (13)	0.0140 (13)
C9D	0.0353 (18)	0.0286 (16)	0.0236 (16)	0.0130 (14)	0.0094 (13)	0.0130 (13)
C10D	0.0299 (16)	0.0243 (15)	0.0194 (15)	0.0059 (12)	0.0065 (12)	0.0091 (12)
C11D	0.0347 (16)	0.0194 (14)	0.0190 (15)	0.0061 (12)	0.0081 (12)	0.0071 (12)
C12D	0.0235 (15)	0.0277 (15)	0.0196 (15)	0.0058 (12)	0.0011 (12)	0.0112 (13)
C13D	0.0227 (15)	0.0350 (17)	0.0176 (15)	0.0047 (13)	0.0012 (12)	0.0084 (13)
C14D	0.0248 (16)	0.0461 (19)	0.0209 (16)	0.0006 (14)	0.0023 (13)	0.0105 (14)
C15D	0.0380 (17)	0.0263 (15)	0.0220 (16)	0.0136 (13)	0.0104 (13)	0.0104 (13)
C16D	0.0371 (17)	0.0258 (15)	0.0218 (16)	−0.0002 (13)	0.0061 (13)	0.0075 (13)
C17D	0.0342 (17)	0.0309 (16)	0.0283 (17)	0.0037 (14)	0.0023 (14)	0.0147 (14)
C18D	0.047 (2)	0.054 (2)	0.0270 (19)	0.0055 (18)	−0.0012 (16)	0.0116 (17)
C19D	0.042 (6)	0.037 (6)	0.040 (5)	0.007 (4)	0.000 (4)	0.007 (4)
C20D	0.035 (5)	0.041 (6)	0.050 (5)	−0.010 (4)	0.005 (4)	0.008 (4)
C21D	0.052 (6)	0.040 (6)	0.025 (4)	−0.002 (5)	0.011 (4)	0.016 (4)
C19X	0.029 (5)	0.046 (7)	0.039 (5)	−0.002 (4)	−0.008 (4)	0.003 (5)
C20X	0.031 (5)	0.045 (6)	0.043 (5)	−0.009 (4)	0.003 (4)	0.021 (4)
C21X	0.029 (5)	0.028 (5)	0.034 (4)	0.006 (3)	0.005 (3)	0.009 (4)
C22D	0.0418 (18)	0.0223 (15)	0.0178 (15)	0.0111 (13)	0.0046 (13)	0.0069 (12)
C23D	0.050 (2)	0.0271 (16)	0.0321 (18)	0.0056 (15)	−0.0063 (15)	0.0144 (15)
C24D	0.061 (2)	0.0307 (18)	0.033 (2)	0.0085 (17)	−0.0130 (17)	0.0042 (16)
C25D	0.072 (3)	0.044 (2)	0.0209 (17)	0.031 (2)	0.0019 (17)	0.0125 (16)
C26D	0.061 (2)	0.046 (2)	0.0260 (18)	0.0286 (19)	0.0171 (17)	0.0195 (16)
C27D	0.0440 (19)	0.0318 (17)	0.0272 (17)	0.0139 (14)	0.0125 (14)	0.0166 (14)

### Geometric parameters (Å, °)

**Table:**

S1A—C12A	1.746 (3)	S1C—C10C	1.837 (3)
S1A—C10A	1.852 (3)	O1C—N2C	1.376 (4)
O1A—N3A	1.359 (3)	O1C—C7C	1.412 (4)
O1A—C7A	1.415 (3)	O2C—C7C	1.208 (4)
O2A—C7A	1.211 (4)	O3C—C9C	1.224 (3)
O3A—C9A	1.216 (3)	O4C—C15C	1.226 (3)
O4A—C15A	1.230 (3)	N1C—N2C	1.295 (4)
N1A—N3A	1.305 (4)	N1C—C8C	1.361 (4)
N1A—C8A	1.364 (4)	N1C—C6C	1.454 (4)
N1A—C6A	1.443 (4)	N3C—N4C	1.415 (3)
N2A—N4A	1.412 (3)	N3C—C11C	1.476 (3)
N2A—C11A	1.474 (4)	N3C—H2NC	0.91 (3)
N2A—H2NA	0.78 (3)	N4C—C12C	1.357 (4)
N4A—C12A	1.354 (3)	N4C—C13C	1.359 (3)
N4A—C13A	1.361 (3)	N5C—C12C	1.312 (4)
N5A—C12A	1.309 (3)	N5C—N6C	1.408 (4)
N5A—N6A	1.394 (3)	N6C—C13C	1.314 (4)
N6A—C13A	1.302 (3)	N7C—C15C	1.343 (4)
N7A—C15A	1.336 (4)	N7C—C14C	1.453 (4)
N7A—C14A	1.454 (4)	N7C—H1NC	0.80 (3)
N7A—H1NA	0.94 (4)	C1C—C6C	1.372 (5)
C1A—C6A	1.378 (10)	C1C—C2C	1.378 (5)
C1A—C2A	1.391 (12)	C1C—H1CA	0.9300
C1A—H1AA	0.9300	C2C—C3C	1.377 (5)
C2A—C3A	1.364 (19)	C2C—H2CA	0.9300
C2A—H2AA	0.9300	C3C—C4C	1.382 (5)
C3A—C4A	1.482 (19)	C3C—H3CA	0.9300
C3A—H3AA	0.9300	C4C—C5C	1.384 (5)
C1X—C2X	1.391 (12)	C4C—H4CA	0.9300
C1X—C6A	1.431 (11)	C5C—C6C	1.372 (4)
C1X—H1XA	0.9300	C5C—H5CA	0.9300
C2X—C3X	1.356 (19)	C7C—C8C	1.408 (5)
C2X—H2XA	0.9300	C8C—C9C	1.453 (4)
C3X—C4A	1.325 (18)	C9C—C10C	1.521 (4)
C3X—H3XA	0.9300	C10C—C11C	1.535 (4)
C4A—C5A	1.379 (5)	C10C—H10C	0.9800
C4A—H4AA	0.9300	C11C—C22C	1.514 (4)
C5A—C6A	1.371 (5)	C11C—H11C	0.9800
C5A—H5AA	0.9300	C13C—C14C	1.480 (4)
C7A—C8A	1.409 (4)	C14C—H14E	0.9700
C8A—C9A	1.463 (4)	C14C—H14F	0.9700
C9A—C10A	1.528 (4)	C15C—C16C	1.495 (4)
C10A—C11A	1.537 (4)	C16C—C21C	1.383 (4)
C10A—H10A	0.9800	C16C—C17C	1.397 (4)
C11A—C22A	1.522 (4)	C17C—C18C	1.381 (4)
C11A—H11A	0.9800	C17C—H17C	0.9300
C13A—C14A	1.491 (4)	C18C—C19C	1.375 (5)
C14A—H14A	0.9700	C18C—H18C	0.9300
C14A—H14B	0.9700	C19C—C20C	1.380 (5)
C15A—C16A	1.501 (4)	C19C—H19C	0.9300
C16A—C17A	1.389 (4)	C20C—C21C	1.371 (4)
C16A—C21A	1.393 (4)	C20C—H20C	0.9300
C17A—C18A	1.384 (4)	C21C—H21C	0.9300
C17A—H17A	0.9300	C22C—C27C	1.384 (4)
C18A—C19A	1.388 (4)	C22C—C23C	1.394 (4)
C18A—H18A	0.9300	C23C—C24C	1.396 (4)
C19A—C20A	1.381 (4)	C23C—H23C	0.9300
C19A—H19A	0.9300	C24C—C25C	1.379 (5)
C20A—C21A	1.378 (4)	C24C—H24C	0.9300
C20A—H20A	0.9300	C25C—C26C	1.377 (5)
C21A—H21A	0.9300	C25C—H25C	0.9300
C22A—C27A	1.386 (4)	C26C—C27C	1.384 (4)
C22A—C23A	1.389 (4)	C26C—H26C	0.9300
C23A—C24A	1.384 (4)	C27C—H27C	0.9300
C23A—H23A	0.9300	S1D—C12D	1.743 (3)
C24A—C25A	1.385 (4)	S1D—C10D	1.839 (3)
C24A—H24A	0.9300	O1D—N2D	1.365 (4)
C25A—C26A	1.373 (4)	O1D—C7D	1.411 (4)
C25A—H25A	0.9300	O2D—C7D	1.208 (4)
C26A—C27A	1.387 (4)	O3D—C9D	1.222 (3)
C26A—H26A	0.9300	O4D—C15D	1.230 (3)
C27A—H27A	0.9300	N1D—N2D	1.305 (3)
S1B—C12B	1.737 (3)	N1D—C8D	1.353 (4)
S1B—C10B	1.855 (3)	N1D—C6D	1.449 (4)
O1B—N2B	1.370 (3)	N3D—N4D	1.417 (3)
O1B—C7B	1.419 (3)	N3D—C11D	1.481 (4)
O2B—C7B	1.208 (4)	N3D—H2ND	0.95 (4)
O3B—C9B	1.215 (3)	N4D—C13D	1.346 (4)
O4B—C15B	1.233 (3)	N4D—C12D	1.361 (3)
N1B—N2B	1.301 (3)	N5D—C12D	1.306 (4)
N1B—C8B	1.364 (4)	N5D—N6D	1.408 (3)
N1B—C6B	1.451 (4)	N6D—C13D	1.317 (4)
N3B—N4B	1.403 (3)	N7D—C15D	1.339 (4)
N3B—C11B	1.491 (4)	N7D—C14D	1.455 (4)
N3B—H2NB	0.88 (3)	N7D—H1ND	0.89 (3)
N4B—C12B	1.358 (3)	C1D—C6D	1.366 (4)
N4B—C13B	1.363 (3)	C1D—C2D	1.379 (5)
N5B—C12B	1.302 (4)	C1D—H1DA	0.9300
N5B—N6B	1.410 (4)	C2D—C3D	1.386 (5)
N6B—C13B	1.298 (4)	C2D—H2DA	0.9300
N7B—C15B	1.346 (4)	C3D—C4D	1.385 (5)
N7B—C14B	1.456 (4)	C3D—H3DA	0.9300
N7B—H1NB	0.86 (3)	C4D—C5D	1.372 (4)
C1B—C2B	1.379 (4)	C4D—H4DA	0.9300
C1B—C6B	1.379 (4)	C5D—C6D	1.391 (4)
C1B—H1BA	0.9300	C5D—H5DA	0.9300
C2B—C3B	1.381 (5)	C7D—C8D	1.412 (4)
C2B—H2BA	0.9300	C8D—C9D	1.452 (4)
C3B—C4B	1.383 (5)	C9D—C10D	1.526 (4)
C3B—H3BA	0.9300	C10D—C11D	1.537 (4)
C4B—C5B	1.384 (5)	C10D—H10D	0.9800
C4B—H4BA	0.9300	C11D—C22D	1.513 (4)
C5B—C6B	1.366 (4)	C11D—H11D	0.9800
C5B—H5BA	0.9300	C13D—C14D	1.485 (4)
C7B—C8B	1.413 (4)	C14D—H14G	0.9700
C8B—C9B	1.449 (4)	C14D—H14H	0.9700
C9B—C10B	1.526 (4)	C15D—C16D	1.481 (4)
C10B—C11B	1.535 (4)	C16D—C17D	1.381 (4)
C10B—H10B	0.9800	C16D—C21X	1.415 (9)
C11B—C22B	1.520 (4)	C16D—C21D	1.419 (10)
C11B—H11B	0.9800	C17D—C18D	1.382 (4)
C13B—C14B	1.480 (4)	C17D—H17D	0.9300
C14B—H14C	0.9700	C18D—C19D	1.330 (11)
C14B—H14D	0.9700	C18D—C19X	1.460 (13)
C15B—C16B	1.492 (4)	C18D—H18D	0.9300
C16B—C17B	1.384 (4)	C19D—C20D	1.381 (14)
C16B—C21B	1.393 (4)	C19D—H19D	0.9300
C17B—C18B	1.383 (4)	C20D—C21D	1.391 (12)
C17B—H17B	0.9300	C20D—H20D	0.9300
C18B—C19B	1.375 (5)	C21D—H21D	0.9300
C18B—H18B	0.9300	C19X—C20X	1.368 (13)
C19B—C20B	1.372 (5)	C19X—H19E	0.9300
C19B—H19B	0.9300	C20X—C21X	1.392 (12)
C20B—C21B	1.382 (5)	C20X—H20E	0.9300
C20B—H20B	0.9300	C21X—H21E	0.9300
C21B—H21B	0.9300	C22D—C23D	1.380 (4)
C22B—C27B	1.385 (4)	C22D—C27D	1.395 (4)
C22B—C23B	1.387 (4)	C23D—C24D	1.396 (4)
C23B—C24B	1.380 (4)	C23D—H23D	0.9300
C23B—H23B	0.9300	C24D—C25D	1.377 (5)
C24B—C25B	1.382 (4)	C24D—H24D	0.9300
C24B—H24B	0.9300	C25D—C26D	1.368 (5)
C25B—C26B	1.372 (4)	C25D—H25D	0.9300
C25B—H25B	0.9300	C26D—C27D	1.370 (4)
C26B—C27B	1.382 (4)	C26D—H26D	0.9300
C26B—H26B	0.9300	C27D—H27D	0.9300
C27B—H27B	0.9300	O1W—H1W1	0.8515
S1C—C12C	1.740 (3)	O1W—H2W1	0.8514
			
C12A—S1A—C10A	99.95 (12)	N2C—O1C—C7C	110.3 (2)
N3A—O1A—C7A	110.6 (2)	N2C—N1C—C8C	114.7 (3)
N3A—N1A—C8A	114.1 (3)	N2C—N1C—C6C	116.4 (3)
N3A—N1A—C6A	116.0 (2)	C8C—N1C—C6C	128.8 (3)
C8A—N1A—C6A	129.7 (3)	N1C—N2C—O1C	105.1 (2)
N4A—N2A—C11A	109.0 (2)	N4C—N3C—C11C	109.9 (2)
N4A—N2A—H2NA	109 (2)	N4C—N3C—H2NC	107.0 (19)
C11A—N2A—H2NA	110 (2)	C11C—N3C—H2NC	105.9 (19)
N1A—N3A—O1A	105.5 (2)	C12C—N4C—C13C	106.1 (2)
C12A—N4A—C13A	105.9 (2)	C12C—N4C—N3C	128.9 (2)
C12A—N4A—N2A	128.2 (2)	C13C—N4C—N3C	124.6 (2)
C13A—N4A—N2A	125.6 (2)	C12C—N5C—N6C	105.9 (2)
C12A—N5A—N6A	106.0 (2)	C13C—N6C—N5C	107.9 (2)
C13A—N6A—N5A	108.4 (2)	C15C—N7C—C14C	120.1 (3)
C15A—N7A—C14A	121.3 (3)	C15C—N7C—H1NC	123 (2)
C15A—N7A—H1NA	122 (2)	C14C—N7C—H1NC	116 (2)
C14A—N7A—H1NA	116 (2)	C6C—C1C—C2C	118.5 (3)
C6A—C1A—C2A	112.8 (8)	C6C—C1C—H1CA	120.8
C6A—C1A—H1AA	123.6	C2C—C1C—H1CA	120.8
C2A—C1A—H1AA	123.6	C3C—C2C—C1C	120.5 (4)
C3A—C2A—C1A	121.7 (10)	C3C—C2C—H2CA	119.8
C3A—C2A—H2AA	119.2	C1C—C2C—H2CA	119.8
C1A—C2A—H2AA	119.2	C2C—C3C—C4C	119.8 (4)
C2A—C3A—C4A	123.4 (11)	C2C—C3C—H3CA	120.1
C2A—C3A—H3AA	118.3	C4C—C3C—H3CA	120.1
C4A—C3A—H3AA	118.3	C3C—C4C—C5C	120.6 (3)
C2X—C1X—C6A	122.1 (7)	C3C—C4C—H4CA	119.7
C2X—C1X—H1XA	119.0	C5C—C4C—H4CA	119.7
C6A—C1X—H1XA	119.0	C6C—C5C—C4C	117.9 (3)
C3X—C2X—C1X	119.8 (10)	C6C—C5C—H5CA	121.0
C3X—C2X—H2XA	120.1	C4C—C5C—H5CA	121.0
C1X—C2X—H2XA	120.1	C1C—C6C—C5C	122.7 (3)
C4A—C3X—C2X	117.4 (13)	C1C—C6C—N1C	118.3 (3)
C4A—C3X—H3XA	121.3	C5C—C6C—N1C	118.9 (3)
C2X—C3X—H3XA	121.3	O2C—C7C—C8C	135.7 (3)
C3X—C4A—C5A	125.2 (8)	O2C—C7C—O1C	120.0 (3)
C3X—C4A—C3A	22.9 (7)	C8C—C7C—O1C	104.3 (3)
C5A—C4A—C3A	113.2 (7)	N1C—C8C—C7C	105.6 (3)
C3X—C4A—H4AA	108.2	N1C—C8C—C9C	124.7 (3)
C5A—C4A—H4AA	123.4	C7C—C8C—C9C	127.8 (3)
C3A—C4A—H4AA	123.4	O3C—C9C—C8C	123.6 (3)
C6A—C5A—C4A	119.2 (3)	O3C—C9C—C10C	122.0 (3)
C6A—C5A—H5AA	120.4	C8C—C9C—C10C	114.4 (3)
C4A—C5A—H5AA	120.4	C9C—C10C—C11C	112.0 (2)
C5A—C6A—C1A	127.7 (5)	C9C—C10C—S1C	108.24 (18)
C5A—C6A—C1X	114.1 (5)	C11C—C10C—S1C	115.31 (18)
C1A—C6A—C1X	32.9 (5)	C9C—C10C—H10C	106.9
C5A—C6A—N1A	118.9 (3)	C11C—C10C—H10C	106.9
C1A—C6A—N1A	110.3 (6)	S1C—C10C—H10C	106.9
C1X—C6A—N1A	125.5 (4)	N3C—C11C—C22C	110.9 (2)
O2A—C7A—C8A	135.7 (3)	N3C—C11C—C10C	113.5 (2)
O2A—C7A—O1A	120.1 (3)	C22C—C11C—C10C	115.5 (2)
C8A—C7A—O1A	104.2 (2)	N3C—C11C—H11C	105.3
N1A—C8A—C7A	105.5 (3)	C22C—C11C—H11C	105.3
N1A—C8A—C9A	126.0 (3)	C10C—C11C—H11C	105.3
C7A—C8A—C9A	128.4 (3)	N5C—C12C—N4C	110.8 (3)
O3A—C9A—C8A	122.8 (3)	N5C—C12C—S1C	124.9 (2)
O3A—C9A—C10A	122.7 (3)	N4C—C12C—S1C	123.9 (2)
C8A—C9A—C10A	114.5 (2)	N6C—C13C—N4C	109.3 (3)
C9A—C10A—C11A	111.6 (2)	N6C—C13C—C14C	126.3 (3)
C9A—C10A—S1A	107.49 (18)	N4C—C13C—C14C	124.3 (3)
C11A—C10A—S1A	114.83 (18)	N7C—C14C—C13C	111.7 (2)
C9A—C10A—H10A	107.5	N7C—C14C—H14E	109.3
C11A—C10A—H10A	107.5	C13C—C14C—H14E	109.3
S1A—C10A—H10A	107.5	N7C—C14C—H14F	109.3
N2A—C11A—C22A	109.3 (2)	C13C—C14C—H14F	109.3
N2A—C11A—C10A	113.9 (2)	H14E—C14C—H14F	107.9
C22A—C11A—C10A	115.4 (2)	O4C—C15C—N7C	122.0 (3)
N2A—C11A—H11A	105.8	O4C—C15C—C16C	121.8 (3)
C22A—C11A—H11A	105.8	N7C—C15C—C16C	116.2 (2)
C10A—C11A—H11A	105.8	C21C—C16C—C17C	120.1 (3)
N5A—C12A—N4A	110.5 (2)	C21C—C16C—C15C	117.6 (3)
N5A—C12A—S1A	124.6 (2)	C17C—C16C—C15C	122.3 (3)
N4A—C12A—S1A	124.68 (19)	C18C—C17C—C16C	118.6 (3)
N6A—C13A—N4A	109.1 (2)	C18C—C17C—H17C	120.7
N6A—C13A—C14A	126.8 (3)	C16C—C17C—H17C	120.7
N4A—C13A—C14A	124.0 (2)	C19C—C18C—C17C	120.6 (3)
N7A—C14A—C13A	112.6 (3)	C19C—C18C—H18C	119.7
N7A—C14A—H14A	109.1	C17C—C18C—H18C	119.7
C13A—C14A—H14A	109.1	C18C—C19C—C20C	120.7 (3)
N7A—C14A—H14B	109.1	C18C—C19C—H19C	119.6
C13A—C14A—H14B	109.1	C20C—C19C—H19C	119.6
H14A—C14A—H14B	107.8	C21C—C20C—C19C	119.2 (3)
O4A—C15A—N7A	121.9 (3)	C21C—C20C—H20C	120.4
O4A—C15A—C16A	121.3 (2)	C19C—C20C—H20C	120.4
N7A—C15A—C16A	116.8 (3)	C20C—C21C—C16C	120.7 (3)
C17A—C16A—C21A	119.5 (3)	C20C—C21C—H21C	119.7
C17A—C16A—C15A	123.4 (3)	C16C—C21C—H21C	119.7
C21A—C16A—C15A	117.1 (3)	C27C—C22C—C23C	118.5 (3)
C18A—C17A—C16A	120.0 (3)	C27C—C22C—C11C	117.7 (3)
C18A—C17A—H17A	120.0	C23C—C22C—C11C	123.7 (3)
C16A—C17A—H17A	120.0	C22C—C23C—C24C	120.2 (3)
C17A—C18A—C19A	120.0 (3)	C22C—C23C—H23C	119.9
C17A—C18A—H18A	120.0	C24C—C23C—H23C	119.9
C19A—C18A—H18A	120.0	C25C—C24C—C23C	120.2 (3)
C20A—C19A—C18A	120.2 (3)	C25C—C24C—H24C	119.9
C20A—C19A—H19A	119.9	C23C—C24C—H24C	119.9
C18A—C19A—H19A	119.9	C26C—C25C—C24C	119.8 (3)
C21A—C20A—C19A	120.0 (3)	C26C—C25C—H25C	120.1
C21A—C20A—H20A	120.0	C24C—C25C—H25C	120.1
C19A—C20A—H20A	120.0	C25C—C26C—C27C	120.2 (3)
C20A—C21A—C16A	120.4 (3)	C25C—C26C—H26C	119.9
C20A—C21A—H21A	119.8	C27C—C26C—H26C	119.9
C16A—C21A—H21A	119.8	C26C—C27C—C22C	121.1 (3)
C27A—C22A—C23A	118.8 (3)	C26C—C27C—H27C	119.4
C27A—C22A—C11A	123.5 (2)	C22C—C27C—H27C	119.4
C23A—C22A—C11A	117.6 (2)	C12D—S1D—C10D	100.30 (13)
C24A—C23A—C22A	120.7 (3)	N2D—O1D—C7D	110.6 (2)
C24A—C23A—H23A	119.7	N2D—N1D—C8D	113.7 (3)
C22A—C23A—H23A	119.7	N2D—N1D—C6D	116.9 (2)
C23A—C24A—C25A	119.9 (3)	C8D—N1D—C6D	129.3 (2)
C23A—C24A—H24A	120.1	N1D—N2D—O1D	105.7 (2)
C25A—C24A—H24A	120.1	N4D—N3D—C11D	109.9 (2)
C26A—C25A—C24A	119.8 (3)	N4D—N3D—H2ND	112 (2)
C26A—C25A—H25A	120.1	C11D—N3D—H2ND	101 (2)
C24A—C25A—H25A	120.1	C13D—N4D—C12D	105.9 (2)
C25A—C26A—C27A	120.4 (3)	C13D—N4D—N3D	125.5 (2)
C25A—C26A—H26A	119.8	C12D—N4D—N3D	128.3 (2)
C27A—C26A—H26A	119.8	C12D—N5D—N6D	106.2 (2)
C22A—C27A—C26A	120.3 (3)	C13D—N6D—N5D	107.2 (2)
C22A—C27A—H27A	119.8	C15D—N7D—C14D	120.3 (2)
C26A—C27A—H27A	119.8	C15D—N7D—H1ND	121.1 (19)
C12B—S1B—C10B	99.92 (12)	C14D—N7D—H1ND	118.6 (19)
N2B—O1B—C7B	110.6 (2)	C6D—C1D—C2D	117.8 (3)
N2B—N1B—C8B	114.6 (2)	C6D—C1D—H1DA	121.1
N2B—N1B—C6B	116.3 (2)	C2D—C1D—H1DA	121.1
C8B—N1B—C6B	128.7 (2)	C1D—C2D—C3D	120.9 (3)
N1B—N2B—O1B	105.2 (2)	C1D—C2D—H2DA	119.5
N4B—N3B—C11B	107.7 (2)	C3D—C2D—H2DA	119.5
N4B—N3B—H2NB	102 (2)	C4D—C3D—C2D	120.0 (4)
C11B—N3B—H2NB	111 (2)	C4D—C3D—H3DA	120.0
C12B—N4B—C13B	105.9 (2)	C2D—C3D—H3DA	120.0
C12B—N4B—N3B	127.2 (2)	C5D—C4D—C3D	119.8 (3)
C13B—N4B—N3B	126.2 (2)	C5D—C4D—H4DA	120.1
C12B—N5B—N6B	106.8 (2)	C3D—C4D—H4DA	120.1
C13B—N6B—N5B	107.2 (2)	C4D—C5D—C6D	118.7 (3)
C15B—N7B—C14B	120.2 (2)	C4D—C5D—H5DA	120.7
C15B—N7B—H1NB	122 (2)	C6D—C5D—H5DA	120.7
C14B—N7B—H1NB	117 (2)	C1D—C6D—C5D	122.7 (3)
C2B—C1B—C6B	117.7 (3)	C1D—C6D—N1D	119.0 (3)
C2B—C1B—H1BA	121.1	C5D—C6D—N1D	118.3 (3)
C6B—C1B—H1BA	121.1	O2D—C7D—O1D	120.2 (3)
C1B—C2B—C3B	120.4 (3)	O2D—C7D—C8D	136.1 (3)
C1B—C2B—H2BA	119.8	O1D—C7D—C8D	103.6 (3)
C3B—C2B—H2BA	119.8	N1D—C8D—C7D	106.4 (2)
C2B—C3B—C4B	120.3 (3)	N1D—C8D—C9D	124.0 (3)
C2B—C3B—H3BA	119.8	C7D—C8D—C9D	129.3 (3)
C4B—C3B—H3BA	119.8	O3D—C9D—C8D	122.3 (3)
C3B—C4B—C5B	120.1 (3)	O3D—C9D—C10D	122.1 (3)
C3B—C4B—H4BA	119.9	C8D—C9D—C10D	115.6 (3)
C5B—C4B—H4BA	119.9	C9D—C10D—C11D	110.2 (2)
C6B—C5B—C4B	118.0 (3)	C9D—C10D—S1D	108.08 (18)
C6B—C5B—H5BA	121.0	C11D—C10D—S1D	115.69 (19)
C4B—C5B—H5BA	121.0	C9D—C10D—H10D	107.5
C5B—C6B—C1B	123.4 (3)	C11D—C10D—H10D	107.5
C5B—C6B—N1B	118.9 (3)	S1D—C10D—H10D	107.5
C1B—C6B—N1B	117.3 (3)	N3D—C11D—C22D	110.1 (2)
O2B—C7B—C8B	135.6 (3)	N3D—C11D—C10D	113.1 (2)
O2B—C7B—O1B	120.5 (3)	C22D—C11D—C10D	115.6 (2)
C8B—C7B—O1B	103.9 (3)	N3D—C11D—H11D	105.7
N1B—C8B—C7B	105.6 (2)	C22D—C11D—H11D	105.7
N1B—C8B—C9B	125.3 (2)	C10D—C11D—H11D	105.7
C7B—C8B—C9B	129.0 (3)	N5D—C12D—N4D	110.7 (2)
O3B—C9B—C8B	121.9 (3)	N5D—C12D—S1D	124.9 (2)
O3B—C9B—C10B	122.4 (2)	N4D—C12D—S1D	124.2 (2)
C8B—C9B—C10B	115.7 (2)	N6D—C13D—N4D	109.9 (2)
C9B—C10B—C11B	111.1 (2)	N6D—C13D—C14D	125.6 (3)
C9B—C10B—S1B	108.09 (18)	N4D—C13D—C14D	124.4 (3)
C11B—C10B—S1B	113.84 (18)	N7D—C14D—C13D	112.0 (2)
C9B—C10B—H10B	107.9	N7D—C14D—H14G	109.2
C11B—C10B—H10B	107.9	C13D—C14D—H14G	109.2
S1B—C10B—H10B	107.9	N7D—C14D—H14H	109.2
N3B—C11B—C22B	108.9 (2)	C13D—C14D—H14H	109.2
N3B—C11B—C10B	112.6 (2)	H14G—C14D—H14H	107.9
C22B—C11B—C10B	115.1 (2)	O4D—C15D—N7D	121.8 (3)
N3B—C11B—H11B	106.6	O4D—C15D—C16D	121.1 (3)
C22B—C11B—H11B	106.6	N7D—C15D—C16D	117.1 (2)
C10B—C11B—H11B	106.6	C17D—C16D—C21X	121.5 (5)
N5B—C12B—N4B	110.1 (2)	C17D—C16D—C21D	113.3 (5)
N5B—C12B—S1B	125.6 (2)	C21X—C16D—C21D	29.4 (4)
N4B—C12B—S1B	124.2 (2)	C17D—C16D—C15D	124.2 (3)
N6B—C13B—N4B	109.9 (2)	C21X—C16D—C15D	111.7 (5)
N6B—C13B—C14B	126.6 (3)	C21D—C16D—C15D	121.3 (4)
N4B—C13B—C14B	123.4 (3)	C16D—C17D—C18D	121.0 (3)
N7B—C14B—C13B	110.8 (2)	C16D—C17D—H17D	119.5
N7B—C14B—H14C	109.5	C18D—C17D—H17D	119.5
C13B—C14B—H14C	109.5	C19D—C18D—C17D	124.0 (6)
N7B—C14B—H14D	109.5	C19D—C18D—C19X	28.6 (4)
C13B—C14B—H14D	109.5	C17D—C18D—C19X	115.3 (5)
H14C—C14B—H14D	108.1	C19D—C18D—H18D	118.0
O4B—C15B—N7B	121.9 (3)	C17D—C18D—H18D	118.0
O4B—C15B—C16B	120.9 (3)	C19X—C18D—H18D	118.6
N7B—C15B—C16B	117.3 (2)	C18D—C19D—C20D	116.5 (9)
C17B—C16B—C21B	119.4 (3)	C18D—C19D—H19D	121.8
C17B—C16B—C15B	123.4 (2)	C20D—C19D—H19D	121.8
C21B—C16B—C15B	117.1 (3)	C19D—C20D—C21D	120.3 (8)
C18B—C17B—C16B	120.1 (3)	C19D—C20D—H20D	119.9
C18B—C17B—H17B	120.0	C21D—C20D—H20D	119.9
C16B—C17B—H17B	120.0	C20D—C21D—C16D	123.0 (7)
C19B—C18B—C17B	119.9 (3)	C20D—C21D—H21D	118.5
C19B—C18B—H18B	120.1	C16D—C21D—H21D	118.5
C17B—C18B—H18B	120.1	C20X—C19X—C18D	122.2 (8)
C20B—C19B—C18B	120.9 (3)	C20X—C19X—H19E	118.9
C20B—C19B—H19B	119.6	C18D—C19X—H19E	118.9
C18B—C19B—H19B	119.6	C19X—C20X—C21X	120.8 (8)
C19B—C20B—C21B	119.6 (3)	C19X—C20X—H20E	119.6
C19B—C20B—H20B	120.2	C21X—C20X—H20E	119.6
C21B—C20B—H20B	120.2	C20X—C21X—C16D	117.0 (7)
C20B—C21B—C16B	120.2 (3)	C20X—C21X—H21E	121.5
C20B—C21B—H21B	119.9	C16D—C21X—H21E	121.5
C16B—C21B—H21B	119.9	C23D—C22D—C27D	118.2 (3)
C27B—C22B—C23B	117.9 (3)	C23D—C22D—C11D	124.9 (3)
C27B—C22B—C11B	123.6 (2)	C27D—C22D—C11D	116.8 (3)
C23B—C22B—C11B	118.4 (2)	C22D—C23D—C24D	120.5 (3)
C24B—C23B—C22B	121.3 (3)	C22D—C23D—H23D	119.7
C24B—C23B—H23B	119.3	C24D—C23D—H23D	119.7
C22B—C23B—H23B	119.3	C25D—C24D—C23D	119.6 (3)
C23B—C24B—C25B	119.8 (3)	C25D—C24D—H24D	120.2
C23B—C24B—H24B	120.1	C23D—C24D—H24D	120.2
C25B—C24B—H24B	120.1	C26D—C25D—C24D	120.4 (3)
C26B—C25B—C24B	119.7 (3)	C26D—C25D—H25D	119.8
C26B—C25B—H25B	120.1	C24D—C25D—H25D	119.8
C24B—C25B—H25B	120.1	C25D—C26D—C27D	119.9 (3)
C25B—C26B—C27B	120.2 (3)	C25D—C26D—H26D	120.0
C25B—C26B—H26B	119.9	C27D—C26D—H26D	120.0
C27B—C26B—H26B	119.9	C26D—C27D—C22D	121.3 (3)
C26B—C27B—C22B	121.0 (3)	C26D—C27D—H27D	119.4
C26B—C27B—H27B	119.5	C22D—C27D—H27D	119.4
C22B—C27B—H27B	119.5	H1W1—O1W—H2W1	125.3
C12C—S1C—C10C	100.08 (13)		
			
C8A—N1A—N3A—O1A	−0.7 (3)	C8C—N1C—N2C—O1C	0.8 (3)
C6A—N1A—N3A—O1A	−176.5 (2)	C6C—N1C—N2C—O1C	178.5 (2)
C7A—O1A—N3A—N1A	1.1 (3)	C7C—O1C—N2C—N1C	−0.8 (3)
C11A—N2A—N4A—C12A	37.1 (4)	C11C—N3C—N4C—C12C	−39.7 (3)
C11A—N2A—N4A—C13A	−136.8 (3)	C11C—N3C—N4C—C13C	148.7 (2)
C12A—N5A—N6A—C13A	1.1 (3)	C12C—N5C—N6C—C13C	−0.3 (3)
C6A—C1A—C2A—C3A	−4.8 (12)	C6C—C1C—C2C—C3C	1.0 (5)
C1A—C2A—C3A—C4A	−2.1 (17)	C1C—C2C—C3C—C4C	−0.5 (5)
C6A—C1X—C2X—C3X	6.0 (19)	C2C—C3C—C4C—C5C	−0.6 (5)
C1X—C2X—C3X—C4A	−0.7 (18)	C3C—C4C—C5C—C6C	1.1 (5)
C2X—C3X—C4A—C5A	4.6 (15)	C2C—C1C—C6C—C5C	−0.4 (5)
C2X—C3X—C4A—C3A	−60 (3)	C2C—C1C—C6C—N1C	179.0 (3)
C2A—C3A—C4A—C3X	127 (4)	C4C—C5C—C6C—C1C	−0.6 (5)
C2A—C3A—C4A—C5A	0.3 (14)	C4C—C5C—C6C—N1C	180.0 (3)
C3X—C4A—C5A—C6A	−13.8 (10)	N2C—N1C—C6C—C1C	−69.2 (4)
C3A—C4A—C5A—C6A	8.6 (7)	C8C—N1C—C6C—C1C	108.0 (3)
C4A—C5A—C6A—C1A	−18.0 (8)	N2C—N1C—C6C—C5C	110.2 (3)
C4A—C5A—C6A—C1X	17.2 (9)	C8C—N1C—C6C—C5C	−72.6 (4)
C4A—C5A—C6A—N1A	−176.2 (3)	N2C—O1C—C7C—O2C	179.6 (3)
C2A—C1A—C6A—C5A	15.4 (9)	N2C—O1C—C7C—C8C	0.4 (3)
C2A—C1A—C6A—C1X	−60.5 (12)	N2C—N1C—C8C—C7C	−0.6 (3)
C2A—C1A—C6A—N1A	175.1 (5)	C6C—N1C—C8C—C7C	−177.8 (3)
C2X—C1X—C6A—C5A	−14.1 (14)	N2C—N1C—C8C—C9C	164.8 (3)
C2X—C1X—C6A—C1A	108.7 (19)	C6C—N1C—C8C—C9C	−12.5 (5)
C2X—C1X—C6A—N1A	−179.7 (8)	O2C—C7C—C8C—N1C	−178.9 (3)
N3A—N1A—C6A—C5A	108.2 (3)	O1C—C7C—C8C—N1C	0.1 (3)
C8A—N1A—C6A—C5A	−66.7 (4)	O2C—C7C—C8C—C9C	16.3 (6)
N3A—N1A—C6A—C1A	−53.5 (5)	O1C—C7C—C8C—C9C	−164.7 (3)
C8A—N1A—C6A—C1A	131.6 (5)	N1C—C8C—C9C—O3C	17.4 (4)
N3A—N1A—C6A—C1X	−86.8 (10)	C7C—C8C—C9C—O3C	179.5 (3)
C8A—N1A—C6A—C1X	98.2 (10)	N1C—C8C—C9C—C10C	−160.6 (3)
N3A—O1A—C7A—O2A	179.9 (2)	C7C—C8C—C9C—C10C	1.4 (4)
N3A—O1A—C7A—C8A	−1.0 (3)	O3C—C9C—C10C—C11C	−28.2 (3)
N3A—N1A—C8A—C7A	0.1 (3)	C8C—C9C—C10C—C11C	149.9 (2)
C6A—N1A—C8A—C7A	175.1 (3)	O3C—C9C—C10C—S1C	100.0 (3)
N3A—N1A—C8A—C9A	−177.8 (3)	C8C—C9C—C10C—S1C	−81.9 (3)
C6A—N1A—C8A—C9A	−2.8 (5)	C12C—S1C—C10C—C9C	−99.4 (2)
O2A—C7A—C8A—N1A	179.5 (3)	C12C—S1C—C10C—C11C	27.0 (2)
O1A—C7A—C8A—N1A	0.6 (3)	N4C—N3C—C11C—C22C	−72.8 (3)
O2A—C7A—C8A—C9A	−2.7 (6)	N4C—N3C—C11C—C10C	59.2 (3)
O1A—C7A—C8A—C9A	178.4 (3)	C9C—C10C—C11C—N3C	68.1 (3)
N1A—C8A—C9A—O3A	−13.6 (4)	S1C—C10C—C11C—N3C	−56.3 (3)
C7A—C8A—C9A—O3A	169.0 (3)	C9C—C10C—C11C—C22C	−162.2 (2)
N1A—C8A—C9A—C10A	167.3 (3)	S1C—C10C—C11C—C22C	73.4 (3)
C7A—C8A—C9A—C10A	−10.2 (4)	N6C—N5C—C12C—N4C	0.0 (3)
O3A—C9A—C10A—C11A	28.4 (3)	N6C—N5C—C12C—S1C	172.69 (19)
C8A—C9A—C10A—C11A	−152.5 (2)	C13C—N4C—C12C—N5C	0.4 (3)
O3A—C9A—C10A—S1A	−98.4 (3)	N3C—N4C—C12C—N5C	−172.4 (2)
C8A—C9A—C10A—S1A	80.8 (2)	C13C—N4C—C12C—S1C	−172.4 (2)
C12A—S1A—C10A—C9A	107.6 (2)	N3C—N4C—C12C—S1C	14.8 (4)
C12A—S1A—C10A—C11A	−17.2 (2)	C10C—S1C—C12C—N5C	−178.7 (2)
N4A—N2A—C11A—C22A	67.7 (3)	C10C—S1C—C12C—N4C	−6.9 (3)
N4A—N2A—C11A—C10A	−63.0 (3)	N5C—N6C—C13C—N4C	0.6 (3)
C9A—C10A—C11A—N2A	−68.3 (3)	N5C—N6C—C13C—C14C	−176.8 (3)
S1A—C10A—C11A—N2A	54.3 (3)	C12C—N4C—C13C—N6C	−0.6 (3)
C9A—C10A—C11A—C22A	164.0 (2)	N3C—N4C—C13C—N6C	172.6 (2)
S1A—C10A—C11A—C22A	−73.4 (3)	C12C—N4C—C13C—C14C	176.8 (2)
N6A—N5A—C12A—N4A	0.3 (3)	N3C—N4C—C13C—C14C	−10.0 (4)
N6A—N5A—C12A—S1A	−174.8 (2)	C15C—N7C—C14C—C13C	−156.7 (3)
C13A—N4A—C12A—N5A	−1.6 (3)	N6C—C13C—C14C—N7C	−107.1 (3)
N2A—N4A—C12A—N5A	−176.4 (2)	N4C—C13C—C14C—N7C	75.9 (3)
C13A—N4A—C12A—S1A	173.5 (2)	C14C—N7C—C15C—O4C	5.8 (4)
N2A—N4A—C12A—S1A	−1.3 (4)	C14C—N7C—C15C—C16C	−174.1 (2)
C10A—S1A—C12A—N5A	166.2 (2)	O4C—C15C—C16C—C21C	−23.4 (4)
C10A—S1A—C12A—N4A	−8.2 (3)	N7C—C15C—C16C—C21C	156.5 (3)
N5A—N6A—C13A—N4A	−2.1 (3)	O4C—C15C—C16C—C17C	157.9 (3)
N5A—N6A—C13A—C14A	175.1 (3)	N7C—C15C—C16C—C17C	−22.2 (4)
C12A—N4A—C13A—N6A	2.3 (3)	C21C—C16C—C17C—C18C	−1.7 (4)
N2A—N4A—C13A—N6A	177.3 (2)	C15C—C16C—C17C—C18C	177.0 (3)
C12A—N4A—C13A—C14A	−175.0 (3)	C16C—C17C—C18C—C19C	−0.2 (5)
N2A—N4A—C13A—C14A	−0.1 (4)	C17C—C18C—C19C—C20C	1.7 (5)
C15A—N7A—C14A—C13A	147.6 (3)	C18C—C19C—C20C—C21C	−1.3 (5)
N6A—C13A—C14A—N7A	119.2 (3)	C19C—C20C—C21C—C16C	−0.6 (5)
N4A—C13A—C14A—N7A	−63.9 (4)	C17C—C16C—C21C—C20C	2.1 (4)
C14A—N7A—C15A—O4A	−3.0 (4)	C15C—C16C—C21C—C20C	−176.7 (3)
C14A—N7A—C15A—C16A	176.7 (2)	N3C—C11C—C22C—C27C	−53.3 (3)
O4A—C15A—C16A—C17A	−158.2 (3)	C10C—C11C—C22C—C27C	175.8 (2)
N7A—C15A—C16A—C17A	22.1 (4)	N3C—C11C—C22C—C23C	131.0 (3)
O4A—C15A—C16A—C21A	20.1 (4)	C10C—C11C—C22C—C23C	0.1 (4)
N7A—C15A—C16A—C21A	−159.5 (3)	C27C—C22C—C23C—C24C	−0.3 (4)
C21A—C16A—C17A—C18A	0.6 (4)	C11C—C22C—C23C—C24C	175.3 (3)
C15A—C16A—C17A—C18A	178.9 (3)	C22C—C23C—C24C—C25C	−0.1 (5)
C16A—C17A—C18A—C19A	0.7 (4)	C23C—C24C—C25C—C26C	0.0 (5)
C17A—C18A—C19A—C20A	−0.5 (5)	C24C—C25C—C26C—C27C	0.6 (5)
C18A—C19A—C20A—C21A	−0.9 (5)	C25C—C26C—C27C—C22C	−1.0 (5)
C19A—C20A—C21A—C16A	2.2 (4)	C23C—C22C—C27C—C26C	0.9 (4)
C17A—C16A—C21A—C20A	−2.0 (4)	C11C—C22C—C27C—C26C	−175.0 (3)
C15A—C16A—C21A—C20A	179.5 (2)	C8D—N1D—N2D—O1D	−1.0 (3)
N2A—C11A—C22A—C27A	−129.5 (3)	C6D—N1D—N2D—O1D	−177.6 (2)
C10A—C11A—C22A—C27A	0.4 (4)	C7D—O1D—N2D—N1D	1.2 (3)
N2A—C11A—C22A—C23A	52.1 (3)	C11D—N3D—N4D—C13D	−146.4 (2)
C10A—C11A—C22A—C23A	−178.0 (2)	C11D—N3D—N4D—C12D	40.0 (3)
C27A—C22A—C23A—C24A	−1.5 (4)	C12D—N5D—N6D—C13D	0.7 (3)
C11A—C22A—C23A—C24A	177.0 (2)	C6D—C1D—C2D—C3D	−1.2 (5)
C22A—C23A—C24A—C25A	0.6 (4)	C1D—C2D—C3D—C4D	1.0 (5)
C23A—C24A—C25A—C26A	0.7 (4)	C2D—C3D—C4D—C5D	0.5 (5)
C24A—C25A—C26A—C27A	−1.0 (4)	C3D—C4D—C5D—C6D	−1.7 (4)
C23A—C22A—C27A—C26A	1.1 (4)	C2D—C1D—C6D—C5D	−0.1 (5)
C11A—C22A—C27A—C26A	−177.3 (2)	C2D—C1D—C6D—N1D	177.5 (3)
C25A—C26A—C27A—C22A	0.2 (4)	C4D—C5D—C6D—C1D	1.6 (4)
C8B—N1B—N2B—O1B	−0.8 (3)	C4D—C5D—C6D—N1D	−176.1 (3)
C6B—N1B—N2B—O1B	−174.5 (2)	N2D—N1D—C6D—C1D	−67.2 (4)
C7B—O1B—N2B—N1B	2.1 (3)	C8D—N1D—C6D—C1D	116.8 (3)
C11B—N3B—N4B—C12B	37.3 (3)	N2D—N1D—C6D—C5D	110.6 (3)
C11B—N3B—N4B—C13B	−132.2 (3)	C8D—N1D—C6D—C5D	−65.4 (4)
C12B—N5B—N6B—C13B	1.5 (3)	N2D—O1D—C7D—O2D	−179.3 (3)
C6B—C1B—C2B—C3B	0.4 (4)	N2D—O1D—C7D—C8D	−0.9 (3)
C1B—C2B—C3B—C4B	−1.4 (5)	N2D—N1D—C8D—C7D	0.5 (3)
C2B—C3B—C4B—C5B	2.2 (6)	C6D—N1D—C8D—C7D	176.6 (3)
C3B—C4B—C5B—C6B	−1.9 (5)	N2D—N1D—C8D—C9D	174.9 (3)
C4B—C5B—C6B—C1B	0.9 (5)	C6D—N1D—C8D—C9D	−9.1 (4)
C4B—C5B—C6B—N1B	174.2 (3)	O2D—C7D—C8D—N1D	178.3 (3)
C2B—C1B—C6B—C5B	−0.1 (4)	O1D—C7D—C8D—N1D	0.2 (3)
C2B—C1B—C6B—N1B	−173.5 (2)	O2D—C7D—C8D—C9D	4.4 (6)
N2B—N1B—C6B—C5B	−60.3 (4)	O1D—C7D—C8D—C9D	−173.7 (3)
C8B—N1B—C6B—C5B	127.1 (3)	N1D—C8D—C9D—O3D	−5.7 (4)
N2B—N1B—C6B—C1B	113.4 (3)	C7D—C8D—C9D—O3D	167.3 (3)
C8B—N1B—C6B—C1B	−59.2 (4)	N1D—C8D—C9D—C10D	176.2 (2)
N2B—O1B—C7B—O2B	178.6 (3)	C7D—C8D—C9D—C10D	−10.8 (4)
N2B—O1B—C7B—C8B	−2.6 (3)	O3D—C9D—C10D—C11D	22.8 (4)
N2B—N1B—C8B—C7B	−0.8 (3)	C8D—C9D—C10D—C11D	−159.2 (2)
C6B—N1B—C8B—C7B	172.0 (3)	O3D—C9D—C10D—S1D	−104.5 (3)
N2B—N1B—C8B—C9B	176.1 (3)	C8D—C9D—C10D—S1D	73.5 (3)
C6B—N1B—C8B—C9B	−11.2 (4)	C12D—S1D—C10D—C9D	100.4 (2)
O2B—C7B—C8B—N1B	−179.4 (3)	C12D—S1D—C10D—C11D	−23.7 (2)
O1B—C7B—C8B—N1B	2.0 (3)	N4D—N3D—C11D—C22D	70.3 (3)
O2B—C7B—C8B—C9B	3.9 (6)	N4D—N3D—C11D—C10D	−60.6 (3)
O1B—C7B—C8B—C9B	−174.7 (3)	C9D—C10D—C11D—N3D	−67.7 (3)
N1B—C8B—C9B—O3B	−11.3 (4)	S1D—C10D—C11D—N3D	55.2 (3)
C7B—C8B—C9B—O3B	164.8 (3)	C9D—C10D—C11D—C22D	164.1 (2)
N1B—C8B—C9B—C10B	170.1 (2)	S1D—C10D—C11D—C22D	−73.0 (3)
C7B—C8B—C9B—C10B	−13.7 (4)	N6D—N5D—C12D—N4D	−0.1 (3)
O3B—C9B—C10B—C11B	22.9 (4)	N6D—N5D—C12D—S1D	−174.59 (19)
C8B—C9B—C10B—C11B	−158.5 (2)	C13D—N4D—C12D—N5D	−0.5 (3)
O3B—C9B—C10B—S1B	−102.7 (3)	N3D—N4D—C12D—N5D	174.1 (2)
C8B—C9B—C10B—S1B	75.9 (3)	C13D—N4D—C12D—S1D	174.1 (2)
C12B—S1B—C10B—C9B	111.58 (19)	N3D—N4D—C12D—S1D	−11.4 (4)
C12B—S1B—C10B—C11B	−12.4 (2)	C10D—S1D—C12D—N5D	176.0 (2)
N4B—N3B—C11B—C22B	61.3 (3)	C10D—S1D—C12D—N4D	2.2 (3)
N4B—N3B—C11B—C10B	−67.6 (3)	N5D—N6D—C13D—N4D	−1.0 (3)
C9B—C10B—C11B—N3B	−67.5 (3)	N5D—N6D—C13D—C14D	178.2 (3)
S1B—C10B—C11B—N3B	54.9 (3)	C12D—N4D—C13D—N6D	0.9 (3)
C9B—C10B—C11B—C22B	166.9 (2)	N3D—N4D—C13D—N6D	−173.9 (2)
S1B—C10B—C11B—C22B	−70.7 (3)	C12D—N4D—C13D—C14D	−178.3 (3)
N6B—N5B—C12B—N4B	−0.6 (3)	N3D—N4D—C13D—C14D	7.0 (4)
N6B—N5B—C12B—S1B	−178.3 (2)	C15D—N7D—C14D—C13D	152.5 (3)
C13B—N4B—C12B—N5B	−0.5 (3)	N6D—C13D—C14D—N7D	106.9 (3)
N3B—N4B—C12B—N5B	−171.7 (2)	N4D—C13D—C14D—N7D	−74.1 (4)
C13B—N4B—C12B—S1B	177.3 (2)	C14D—N7D—C15D—O4D	−1.0 (4)
N3B—N4B—C12B—S1B	6.0 (4)	C14D—N7D—C15D—C16D	177.7 (3)
C10B—S1B—C12B—N5B	160.1 (2)	O4D—C15D—C16D—C17D	−159.1 (3)
C10B—S1B—C12B—N4B	−17.3 (3)	N7D—C15D—C16D—C17D	22.2 (4)
N5B—N6B—C13B—N4B	−1.8 (3)	O4D—C15D—C16D—C21X	3.0 (6)
N5B—N6B—C13B—C14B	−179.0 (3)	N7D—C15D—C16D—C21X	−175.7 (6)
C12B—N4B—C13B—N6B	1.4 (3)	O4D—C15D—C16D—C21D	34.2 (8)
N3B—N4B—C13B—N6B	172.8 (2)	N7D—C15D—C16D—C21D	−144.5 (8)
C12B—N4B—C13B—C14B	178.7 (3)	C21X—C16D—C17D—C18D	17.9 (7)
N3B—N4B—C13B—C14B	−9.9 (4)	C21D—C16D—C17D—C18D	−14.1 (8)
C15B—N7B—C14B—C13B	155.4 (3)	C15D—C16D—C17D—C18D	178.3 (3)
N6B—C13B—C14B—N7B	112.6 (3)	C16D—C17D—C18D—C19D	17.4 (9)
N4B—C13B—C14B—N7B	−64.2 (4)	C16D—C17D—C18D—C19X	−14.2 (8)
C14B—N7B—C15B—O4B	1.8 (4)	C17D—C18D—C19D—C20D	−10.5 (12)
C14B—N7B—C15B—C16B	−176.7 (2)	C19X—C18D—C19D—C20D	70.4 (16)
O4B—C15B—C16B—C17B	−153.5 (3)	C18D—C19D—C20D—C21D	2.3 (14)
N7B—C15B—C16B—C17B	25.1 (4)	C19D—C20D—C21D—C16D	−0.8 (15)
O4B—C15B—C16B—C21B	22.7 (4)	C17D—C16D—C21D—C20D	6.5 (12)
N7B—C15B—C16B—C21B	−158.8 (3)	C21X—C16D—C21D—C20D	−106.8 (17)
C21B—C16B—C17B—C18B	−0.4 (4)	C15D—C16D—C21D—C20D	174.5 (7)
C15B—C16B—C17B—C18B	175.7 (3)	C19D—C18D—C19X—C20X	−108 (2)
C16B—C17B—C18B—C19B	0.7 (5)	C17D—C18D—C19X—C20X	6.9 (13)
C17B—C18B—C19B—C20B	−0.3 (5)	C18D—C19X—C20X—C21X	−2.7 (16)
C18B—C19B—C20B—C21B	−0.5 (6)	C19X—C20X—C21X—C16D	5.3 (13)
C19B—C20B—C21B—C16B	0.9 (5)	C17D—C16D—C21X—C20X	−12.7 (10)
C17B—C16B—C21B—C20B	−0.5 (5)	C21D—C16D—C21X—C20X	69.1 (12)
C15B—C16B—C21B—C20B	−176.7 (3)	C15D—C16D—C21X—C20X	−175.3 (6)
N3B—C11B—C22B—C27B	−130.5 (3)	N3D—C11D—C22D—C23D	−126.5 (3)
C10B—C11B—C22B—C27B	−3.0 (4)	C10D—C11D—C22D—C23D	3.2 (4)
N3B—C11B—C22B—C23B	48.9 (3)	N3D—C11D—C22D—C27D	56.1 (3)
C10B—C11B—C22B—C23B	176.5 (2)	C10D—C11D—C22D—C27D	−174.3 (2)
C27B—C22B—C23B—C24B	−0.5 (4)	C27D—C22D—C23D—C24D	0.5 (5)
C11B—C22B—C23B—C24B	−179.9 (3)	C11D—C22D—C23D—C24D	−176.9 (3)
C22B—C23B—C24B—C25B	−0.2 (4)	C22D—C23D—C24D—C25D	0.2 (5)
C23B—C24B—C25B—C26B	0.4 (5)	C23D—C24D—C25D—C26D	−0.1 (5)
C24B—C25B—C26B—C27B	0.1 (5)	C24D—C25D—C26D—C27D	−0.8 (5)
C25B—C26B—C27B—C22B	−0.8 (5)	C25D—C26D—C27D—C22D	1.6 (5)
C23B—C22B—C27B—C26B	0.9 (4)	C23D—C22D—C27D—C26D	−1.4 (4)
C11B—C22B—C27B—C26B	−179.6 (3)	C11D—C22D—C27D—C26D	176.2 (3)

### Hydrogen-bond geometry (Å, °)

**Table:**

*D*—H···*A*	*D*—H	H···*A*	*D*···*A*	*D*—H···*A*
O1W—H2W1···O2C	0.85	1.92	2.772 (4)	175
O1W—H1W1···N6B	0.85	2.10	2.944 (4)	174
N7A—H1NA···N5B^i^	0.94 (5)	2.15 (5)	3.017 (4)	153 (4)
N7A—H1NA···N6B^i^	0.94 (5)	2.19 (6)	3.016 (4)	146 (3)
N2A—H2NA···O3A	0.78 (3)	2.35 (3)	2.912 (3)	130 (3)
N7B—H1NB···N6A^ii^	0.86 (4)	2.16 (4)	2.964 (4)	155 (3)
N3B—H2NB···O3B	0.88 (3)	2.25 (3)	2.865 (3)	127 (3)
N7C—H1NC···N6D^iii^	0.80 (3)	2.21 (3)	2.990 (4)	165 (3)
N3C—H2NC···O3C	0.91 (3)	2.21 (3)	2.895 (3)	132 (3)
N7D—H1ND···N6C^iii^	0.89 (3)	2.25 (3)	3.082 (4)	157 (3)
N3D—H2ND···O3D	0.94 (3)	2.07 (3)	2.841 (3)	138 (3)
C1A—H1XA···O2B^i^	1.16	2.43	3.471 (10)	148
C5A—H5AA···O4A^iv^	0.93	2.45	3.294 (4)	151
C1B—H1BA···O4B^v^	0.93	2.40	3.278 (4)	158
C11A—H11A···O4A^iv^	0.98	2.60	3.291 (4)	128
C5B—H5BA···O2A^ii^	0.93	2.51	3.405 (4)	162
C19D—H19D···O2B^vi^	0.93	2.41	3.289 (11)	157
C5D—H5DA···O4D^iii^	0.93	2.47	3.328 (4)	154
C23A—H23A···O3A^iv^	0.93	2.59	3.318 (4)	136
C23B—H23B···O3B^v^	0.93	2.41	3.240 (4)	149
C27C—H27C···O3C^vii^	0.93	2.52	3.359 (4)	150
C27D—H27D···O3D^iii^	0.93	2.42	3.258 (4)	149

Symmetry codes: (i) *x*+1, *y*, *z*; (ii) *x*−1, *y*, *z*; (iii) −*x*+1, −*y*+1, −*z*; (iv) −*x*+2, −*y*, −*z*+1; (v) −*x*, −*y*+1, −*z*+1; (vi) *x*, *y*, *z*−1; (vii) −*x*+1, −*y*, −*z*.
